# High-Quality Genome of the Medicinal Plant *Strobilanthes cusia* Provides Insights Into the Biosynthesis of Indole Alkaloids

**DOI:** 10.3389/fpls.2021.742420

**Published:** 2021-09-30

**Authors:** Yongle Hu, Dongna Ma, Shuju Ning, Qi Ye, Xuanxuan Zhao, Qiansu Ding, Pingping Liang, Guoqian Cai, Xiaomao Ma, Xia Qin, Daozhi Wei

**Affiliations:** ^1^College of Life Sciences, Fujian Agriculture and Forestry University, Fuzhou, China; ^2^College of Ecology and Resource Engineering, Wuyi University, Wuyishan, China; ^3^Fujian Provincial Key Laboratory of Eco-Industrial Green Technology, Wuyishan, China; ^4^Key Laboratory of the Ministry of Education for Coastal and Wetland Ecosystems, College of the Environment and Ecology, Xiamen University, Xiamen, China; ^5^College of Agriculture, Fujian Agriculture and Forestry University, Fuzhou, China

**Keywords:** *Strobilanthes cusia*, medicinal plant, whole-genome sequencing, lineage-specific genes, basic helix-loop-helix, indole alkaloid biosynthesis

## Abstract

*Strobilanthes cusia* (Nees) Kuntze is an important plant used to process the traditional Chinese herbal medicines “Qingdai” and “Nanbanlangen”. The key active ingredients are indole alkaloids (IAs) that exert antibacterial, antiviral, and antitumor pharmacological activities and serve as natural dyes. We assembled the *S. cusia* genome at the chromosome level through combined PacBio circular consensus sequencing (CCS) and Hi-C sequencing data. Hi-C data revealed a draft genome size of 913.74 Mb, with 904.18 Mb contigs anchored into 16 pseudo-chromosomes. Contig N50 and scaffold N50 were 35.59 and 68.44 Mb, respectively. Of the 32,974 predicted protein-coding genes, 96.52% were functionally annotated in public databases. We predicted 675.66 Mb repetitive sequences, 47.08% of sequences were long terminal repeat (LTR) retrotransposons. Moreover, 983 *Strobilanthes*-specific genes (SSGs) were identified for the first time, accounting for ~2.98% of all protein-coding genes. Further, 245 putative centromeric and 29 putative telomeric fragments were identified. The transcriptome analysis identified 2,975 differentially expressed genes (DEGs) enriched in phenylpropanoid, flavonoid, and triterpenoid biosynthesis. This systematic characterization of key enzyme-coding genes associated with the IA pathway and basic helix-loop-helix (bHLH) transcription factor family formed a network from the shikimate pathway to the indole alkaloid synthesis pathway in *S. cusia*. The high-quality *S. cusia* genome presented herein is an essential resource for the traditional Chinese medicine genomics studies and understanding the genetic underpinning of IA biosynthesis.

## Introduction

*Strobilanthes cusia* (Nees) Kuntze (2*n* = 32) is a perennial dicotyledonous herb of the order Acanthaceae and is broadly distributed from South to East Asia, the countries such as, India, Bangladesh, Thailand, Bhutan, and China (Hu et al., 2011). Generally, *S. cusia* grows in clay or moist soil in mountainous areas and is suitable for transplanting because of its ability to tolerate different soils. As an important medicinal plant and natural dye, *S. cusia* has been cultivated and processed in China for thousands of years (Lin et al., 2018). For instance, the stems and leaves of *S. cusia* are regularly processed to obtain Qingdai (Indigo Naturalis), whereas the dried rhizomes and roots are known as Nanbanlangen (Rhizoma et Radix Baphicacanthis Cusiae), both of which are listed in the Chinese Pharmacopoeia as traditional medicines (Chinese Pharmacopoeia Committee, 2020). The medicinal uses of Qingdai include the treatment of various inflammatory diseases, oral ulcers, and skin diseases. Nanbanlangen has demonstrated efficacy in preventing and treating influenza A infection, mumps, and other infectious diseases (Tanaka et al., 2004; Yu et al., 2021). The phytochemical analyses showed that *S. cusia* can produce high quantities of biologically active compounds, such as, indole alkaloids (IAs), quinolone alkaloids, phenylethanoid glycosides, lignan glycosides, triterpenoids, steroids, amino acids, and flavonoids (Gu et al., 2014; Xiao et al., 2018; Yu et al., 2021). Among these chemical components, indigo and indirubin are the major medicinal ingredients and are isomers of each other (C_16_H_10_N_2_O_2_). The Chinese Pharmacopoeia stipulates that the mass fractions of indigo and indirubin in Qingdai should be higher than 2.0 and 0.13%, respectively, and indigo and indirubin are used as the criteria for identifying Nanbanlangen (Chinese Pharmacopoeia Committee, 2020). Further, clinical and pharmacological evidence suggests that the main alkaloids can treat leukemia (Wang et al., 2008; Wu et al., 2016) and dermatoses (Hsieh et al., 2012); protect against tissue damage (Huang et al., 2017); and exert anti-inflammatory (Sugimoto et al., 2016; Kawai et al., 2017), antibacterial (Chiang et al., 2013; Tsai et al., 2020), and immune-regulatory effects (Zhang et al., 2015; Jie et al., 2017).

Despite the medical importance of *S. cusia*, previous research has mainly focused on its chemistry and pharmacology, whereas research of the active ingredients remains limited. The biosynthetic pathway of the primary medicinal ingredient of *S. cusia*, IAs, remains largely unknown. The previous transcriptomic analyses revealed that cytochrome P450, uridine diphosphate-glycosyltransferase, β-glucosidase, and tryptophan synthase participate in the biosynthesis of indigo and the indole glycoside backbone (Lin et al., 2018, 2019; Xu et al., 2020). Additionally, the enzyme analysis and overexpression experiments confirmed that 5-enolpyruvylshikimate-3-phosphate synthase (*EPSPS*) contributes to the regulation of indigo synthesis (Yu et al., 2019). Additional key enzymes and transcription factors (TFs) involved in synthesizing IAs have not been thoroughly investigated in the *S. cusia* genome.

Owing to its long history of herbal use, wide distribution, and efficacy, *S. cusia* is in high demand in developing countries (Pal and Shukla, 2003; Yu et al., 2021). The Herbs Genome Program is a whole-genome sequencing and post-genomics study of medicinal plants with economically important and characteristic secondary metabolic pathways. This program aimed to characterize the genetic information and regulatory networks of plants used as traditional Chinese medicines through genome sequencing and determine the biosynthetic pathways of the active ingredients in these medicines and the mechanisms underlying the prevention of human diseases (Chen et al., 2015; Hu et al., 2019). To date, genomic data for *Panax ginseng* (Xu et al., 2017), *Scutellaria baicalensis* (Zhao et al., 2019), *Andrographis paniculata* (Sun et al., 2019), and many herbs have been obtained through high-throughput sequencing, and abundant gene information related to the biological evolution, growth and development, stress resistance, and secondary metabolism have been obtained by integrating transcriptome, metabolomic, and proteomics approaches. Thus, the construction of a high-quality *S. cusia* genome will serve to identify, or supplement, the candidate genes associated with the biosynthesis pathway of bioactive compounds and reveal the molecular mechanisms underlying its documented medicinal value.

Although a reference genome sequence was previously published for *S. cusia* (MinION), the assembled long reads were generated using the Oxford Nanopore Technologies platform (Xu et al., 2020). This platform has certain advantages, such as, the generation of longer reads through high-throughput sequencing; however, it also has a moderately high associated error rate, leading to lower assembly accuracy. Thus, in the present study, we assembled high-fidelity reads using the latest PacBio circular consensus sequencing (CCS) technology, which combines both long read length and high accuracy. The CCS sequencing results easily span the shorter repetitive complex regions of the genome while ensuring the precision and completeness of genome assembly (Wenger et al., 2019). In this study, we present the high-quality chromosome-level genome assembly and systematic analysis of species-specific genes, the basic helix-loop-helix (bHLH) family, and the IA pathway in the *S. cusia* genome. Our results provide an important resource for future studies on the mechanisms of active medicinal ingredient synthesis in *S. cusia*.

## Materials and Methods

### Sample Collection and Genome Sequencing

We collected fresh leaves of *S. cusia* for genomic sequencing from our experimental field at the Fujian Agriculture and Forestry University (26°08′ E, 119°23′ N). The leaves were stored at −80°C until DNA extraction using the cetyltrimethylammonium bromide method.

An Illumina paired-end genomic library (average insert size of 350 bp) was constructed according to the standard protocols of Illumina and sequenced on an Illumina HiSeq X Ten platform (San Diego, CA, USA). Library construction (15-kb DNA SMRTbell library) and long-read sequencing were performed using the PacBio CCS technology platform (Menlo Park, CA, USA). We extracted tender leaves to validate the cell fixation and observed the integrity of the nuclei through DAPI staining. In addition, a Hi-C library was created using the *Hin*dIII enzyme according to the BioMarker Technologies Company instructions (Rohnert Park, CA, USA). The sequencing was performed on the Illumina HiSeq X Ten platform. All the sequencing services were provided by Biomarker Technologies Co., Ltd. (Beijing, China).

### Genome Survey and Assembly

GenomeScope in conjunction with Jellyfish (version 2.2.3) (Vurture et al., 2017) was used for genome size estimation of *S. cusia*. In total, 49.98 Gb of high-quality paired-end reads were generated by Illumina genomic sequencing (~52.73 × coverage, Supplementary Table 1). To evaluate the genome size, repeat content, and heterozygosity of *S. cusia*, the k-mer distribution with 21 nt was constructed using clean Illumina short-read data. For PacBio sequencing data, low-quality, joints, and short read filtering of the raw data yielded 483.97 Gb of sub-reads (~532×, Supplementary Table 1), which were assembled using hifiasm (Cheng et al., 2021). Burrows-Wheeler Aligner (BWA; version 0.7.10-r789, a software package) (Li, 2013) and SAMtools (version 1.9) (Li et al., 2009) were used for assembly statistics. Finally, we used the BUSCO (version 3.0) (Simão et al., 2015) database embryophyta_odb10 models to assess the genome integrity.

### Chromosome Assembly Using Hi-C

The Hi-C data (~106.27 Gb) generated were mapped back to the draft assembly using BWA software, and a PERL script developed by LACHESIS software was used to obtain clean data for the Hi-C (Burton et al., 2013). Only mapping data were retained for linkage to pseudo-chromosomes using the ALLHiC pipeline (Zhang et al., 2019). The HiC-Pro (version 2.10.0) program was used to determine the Hi-C mapping rate and for quality assessment (Servant et al., 2015).

### Protein-Coding Gene Prediction

Protein-coding genes were predicted using the three approaches: *ab initio* gene prediction, transcript evidence, and homologous-based analyses. The MAKER pipeline (Cantarel et al., 2007), which integrates all three approaches, was used. For homology-based analysis, we downloaded proteomes homologous to *S. cusia*, such as, *Olea europaea* var. *sylvestris, A. paniculata, Mimulus guttatus, Antirrhinum majus, Salvia splendens, Sesamum indicum*, and *Handroanthus impetiginosus*. The protein sequences from each species were then paired with the *S. cusia* genome using TBLASTN software, and the gene structure was predicted using GeneWise software.

For transcript evidence, we used Scallop (version 0.10.4) (Shao and Kingsford, 2017) software and assembled the RNA-seq samples (stems, roots, and leaves). The transcripts obtained were used for training programs of SNAP (version 2006-07-28) (Bromberg and Rost, 2007), GENEMARK (version 4.48_3.60_lic) (Besemer et al., 2001), and AUGUSTUS (version 3.3.3) (Stanke et al., 2004). The MAKER pipeline was used to integrate these layers of coding evidence to generate predictions of high-quality protein-coding genes.

### Functional Annotation

Functional annotation of the protein-coding genes in *S. cusia* using BLASTP (*E*-value ≤ 1e-5) were aligned with the public databases NR, Swiss-Prot (Bairoch and Apweiler, 2000), Pfam (Finn et al., 2016), Gene Ontology (GO) (Ashburner et al., 2000), Clusters of Orthologous Groups (COG) (Tatusov et al., 2000), and Kyoto Encyclopedia of Genes and Genomes (KEGG) (Kanehisa et al., 2013). A Pfam database search, using PfamScan, identified the protein domain. The online platform OmicShare (https://www.omicshare.com/) was used for the GO enrichment and KEGG pathway analyses. The TFs were predicted using iTAK software, and tRNAscan-SE (version 1.3.1) with eukaryotic parameters (Chan and Lowe, 2019) was used to identify transfer RNA (tRNA) genes; ribosomal RNA (rRNA) fragments were predicted using RNAmmer (version 1.2) (Lagesen et al., 2007). For small nuclear RNA (snRNA) and microRNA (miRNA) gene analysis, INFERNAL (version 1.1.3) with default parameters was used for alignment with the Rfam database (Jones et al., 2014).

### Analysis of Repetitive Elements and Synteny

For *de novo* prediction, we used the RepeatModeler pipeline (Flynn et al., 2020) to customize the repeated sequence library of the genome, which utilizes RECON and RepeatScout to obtain the consensus repeat library. RepeatMasker was further used in a homology-based method to identify and cluster repetitive elements (Tarailo-Graovac and Chen, 2009). A TEclass software was employed to classify unknown transposable element (TE) types (Abrusán et al., 2009). To identify tandem repeated sequences in the *S. cusia* genome, the Finder package was used to determine higher-order repeated sequences (Benson, 1999). The distribution of tandem repeats on the chromosomes was used to predict centromeres and telomeres in the same manner as in the *Oropetium thomaeum* genome (VanBuren et al., 2015). We downloaded the previously published fasta and hic.gff files of the *S. cusia* genome from http://indigoid-plant.iflora.cn. The syntenic gene pairs were identified and plotted by MCScan (Python version) [https://github.com/tanghaibao/jcvi/wiki/MCscan-(Python-version)].

### Phylogenetic and Whole-Genome Duplication (WGD) Analyses

To investigate the evolution of *S. cusia*, we compared its genome with 12 other sequenced plant species. These included one other plant in Acanthaceae (*A. paniculata*), six others in the order Lamiales (*M. guttatus, S. splendens, H. impetiginosus, S. indicum, A. majus*, and *O. europaea*), four others in the clade Eudicots (*Solanum tuberosum, Solanum lycopersicum, Populus trichocarpa*, and *Vitis vinifera*), and *Oryza sativa* as an outgroup. Single-copy orthologous genes were identified by OrthoFinder (v2.3.3) (Emms and Kelly, 2015). MAFFT (v6.864b) with default parameters was used to align each single-copy gene (Katoh and Standley, 2013). The conserved sites were extracted by filtering the alignment with in-house Python scripts. We then strung them together into a unique super sequence. IQ-TREE (v1.7-beta12) (Nguyen et al., 2015) with default parameters was used to predict the best substitution structure models, and maximum likelihood gene trees were constructed by RAxML (Stamatakis, 2014) with 500 bootstraps, with *O. sativa* chosen as an outgroup. For the selected species, we estimated the divergence time. First, we set two fossil constraint divergence times from the TimeTree database (Kumar et al., 2017). *V. vinifera* and *O. sativa* were estimated to have diverged ~160 million years ago (Mya), whereas *S. indicum* and *P. trichocarpa* diverged ~117 Mya. Using these two nodes as fossil times, the divergence time of all plants was evaluated using r8s software (v1.83) (Sanderson, 2003). CAFE (v3.1) (De Bie et al., 2006) was employed to infer the expansion and contraction of the gene family with a *P*-value < 0.01 based on phylogeny.

We performed a BLASTP all-to-all search to identify the homologous genes with an *E*-value ≤ 1E-8 by examining WGD in *S. cusia* and *A. paniculata*. Collinearity blocks with the MCScanX (https://github.com/tanghaibao/jcvi/wiki/) were identified. Next, the synonymous substitution rates (*Ks*) of collinear orthologous gene pairs were calculated with Python script synonymous_calc.py (https://github.com/tanghaibao/bio-pipeline/) using the Nei-Gojobori method (Nei and Gojobori, 1986).

### Transcriptome Analysis

As a vital cellular regulator, methyl jasmonate (MeJA) plays a role in inducing signal transduction during secondary metabolism and can change the metabolites in plants by promoting or inhibiting gene expression. Previously, we reported that the content of indigo in MeJA-treated leaves and roots of *S. cusia* was higher than that in the control group (Lin et al., 2019). The transcriptome data of *S. cusia* accessions were imported from our previous study and filtered using Trimmomatic (Bolger et al., 2014). The treated groups (BL: treated leaf, BS: treated stem, and BR: treated root) were sprayed with 0.01% (v/v) Tween 20 solution containing 22.29 μM MeJA (Sigma-Aldrich, St. Louis, MO, USA), whereas the control groups (AL: control leaf, AS: control stem, and AR: control root) were treated with 0.01% (v/v) Tween 20 solution without MeJA to the point of runoff. Three independent biological replicates were prepared for RNA sequencing. The predicted coding sequences (CDS) of the *S. cusia* genome were then mapped, and the fragments per kilobase of exon per million mapped reads of each gene were calculated using the RSEM (version 1.3.2) program (https://github.com/deweylab/RSEM). The DESeq2 package was used to identify differentially expressed genes (DEGs). The *p*-values were adjusted using Benjamin-Hochberg multiple testing corrections, and the genes were considered as differentially expressed if the false discovery rate was <0.05 and |log2 fold-change| >1.

### Identification and Analysis of Lineage-Specific Genes

The lineage-specific genes (LSGs) have no significant similarity to any sequence in other species (Fischer and Eisenberg, 1999). The plant traits are mainly determined by genes, and adaptation of species to the environment is the main driving force for LSG evolution. We used a comparative genomics approach to identify LSGs in the *S. cusia* genome (Chen et al., 2020). First, we used BLASTP (*E*-value < 1e-3) to compare the proteomic data collected from seven closely related plant species: *O. europaea* var. *sylvestris, A. paniculata, M. guttatus, A. majus, S. splendens, S. indicum*, and *H. impetiginosus*. Homologous sequences were filtered out, and homology searches were conducted using the Plant-PUTs (http://www.plantgdb.org/prj/ESTCluster/progress.php), Phytozome (http://phytozome.jgi.doe.gov/pz/portal.html), UniProt-KB (ftp://ftp.ebi.ac.uk/pub/databases/uniprot/knowledgebase/), and NR databases. Finally, the genes with no homology to any databases were classified as LSGs, whereas, the remaining genes with similarity were classified as evolutionarily conserved genes (ECs). Gene duplication is the primary mechanism of origin. ECs were searched using BLASTP (*E*-value < 1e-3) to infer the paralogs of the LSGs and define hit sequences as gene duplications. The isoelectric points of the LSGs and ECs were calculated using DAMBE software (Xia, 2018), and subcellular localization was evaluated using BUSCO software (Savojardo et al., 2018). The guanine plus cytosine (GC) content, protein length, and exon number of LSGs and ECs were calculated using Python scripts. One-way ANOVA was used to establish any significant differences between the LSGs and ECs.

### Gene Family Analysis of bHLH

The bHLH TFs are important members of the plant gene regulatory network. In addition to participating in the growth and development and responding to adversity stress, bHLH TFs can also play a role in the process of biosynthesis (Jia et al., 2021; Singh et al., 2021). To identify and analyze *S. cusia* at the genome-wide level, we downloaded the protein sequence of bHLHs from *Arabidopsis thaliana* (https://www.arabidopsis.org/browse/genefamily/bHLH.jsp). BLASTP (*E*-value < 1e-5) was performed to obtain the potential bHLHs, whereas SMART (http://smart.embl-heidelberg.de/) was used to predict the conserved structural domains and obtain high-quality bHLHs. The essential physicochemical characteristics of bHLHs, such as, their amino acid number, molecular weight, and isoelectric point, were predicted using DAMBE. MEME (https://meme-suite.org/meme/index.html) was utilized for the enrichment analysis of the conserved structural domains. Finally, a phylogenetic tree with 1,000 bootstrap replicates was constructed using the MEGA 7 program with the neighbor-joining method (Kumar et al., 2016).

## Results

### Genome Assembly

We estimated the genome size of *S. cusia* by 21 k-mer counting's. According to the k-mer distribution (Supplementary Figure 1), the length of the genome was ~947.79 Mb with heterozygosity of 0.45%, repeat content of 78.84%, and GC content of 38.96%. We generated 483.97 Gb (532×) of PacBio long CCS reads and ~49.98 Gb (53×) of Illumina clean reads (Supplementary Table 1) and used hifiasm to assemble the genome with 913.74 Mb that includes 1,357 contigs (N50 size of 35.59 Mb) with the longest contig of 66.79 Mb (Table 1). The initial assembly result was ~8-fold more contiguous than the other released version (MinION), where the N50 size of contigs was 4.33 Mb. We detected genic completeness using BUSCO (97.8%), of which 92.3% were single-copy genes and 5.5% were duplicated (Supplementary Table 2), which was slightly higher than that in MinION (88.12%). Additionally, we used Illumina read mapping for assembly with BWA-MEN and showed that ~49.91 Gb of reads was available for mapping on the genome, with a 99.85% mapping rate (Supplementary Table 3).

**Table 1:** Global statistics for assembly and annotation of *Strobilanthes cusia* genome.

**Items**	**MinION**	**PacBio CCS**
**Sequencing assembly**		
Estimated genome size (Mb)	826.37	947.79
Contig size (Mb)	865.49	913.74
Number of contigs	1602	1357
Contig N50 (Mb)	4.33	35.59
Longest contig (Mb)	20.84	66.79
BUSCO completeness of assembly (%)	88.12	97.8
**Hi-C scaffolding assembly**		
Scaffold size (Mb)	865.52	912.62
Number of scaffolds	1354	108
Scaffold N50 (Mb)	50.44	68.44
Average length (Mb)	0.64	8.45
**Gene annotation**		
Protein-coding genes number	32148	32974
Average gene length (bp)	3450.12	3399.37
Average exon length (bp)	244.48	310.24
Average exon per gene	5.23	6.01
Average intron length (bp)	302.2	306.51
BUSCO completeness of annotation (%)	91.39	98.5

**Figure 1:**
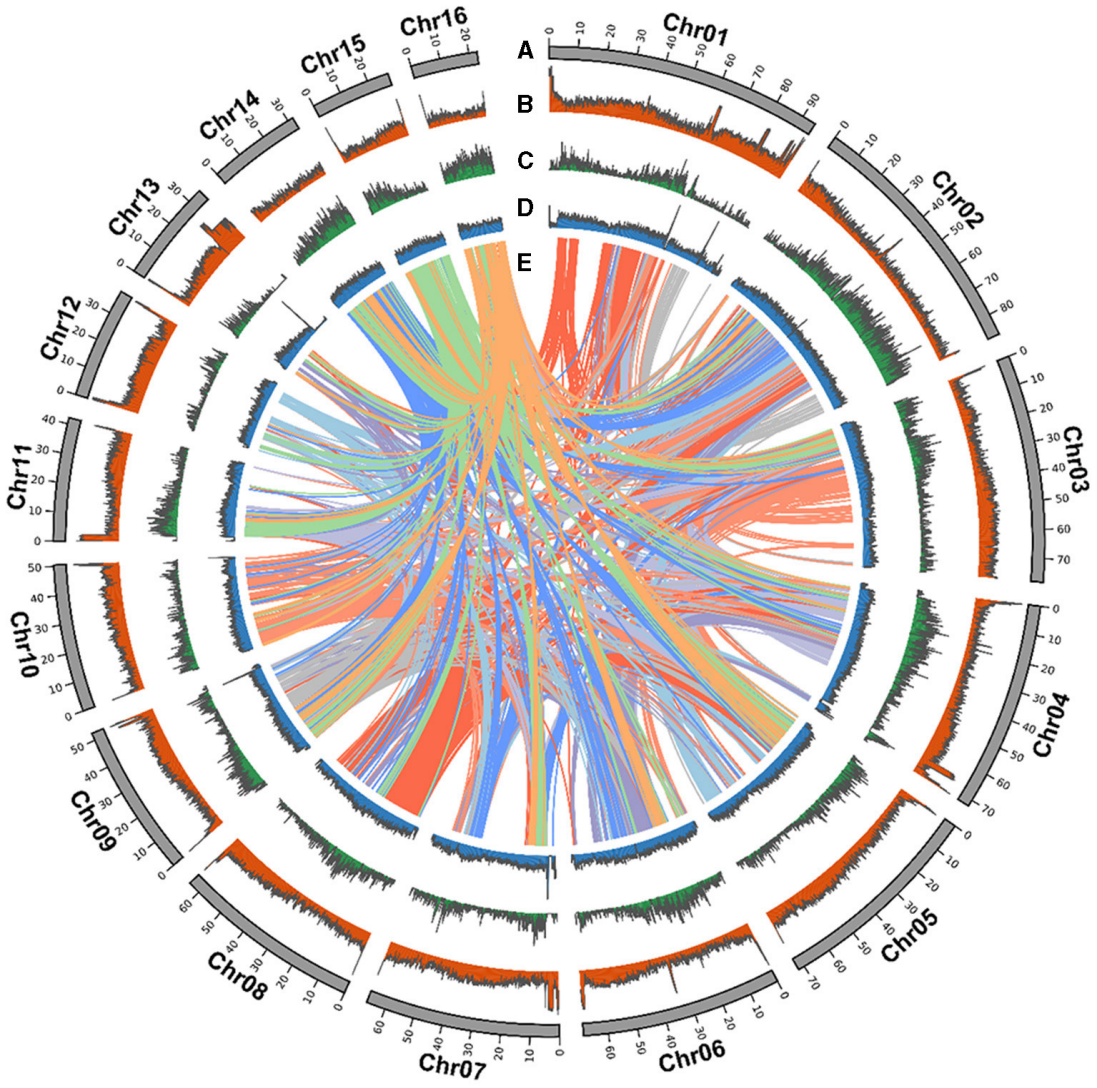
*Strobilanthes cusia* chromosomal elements in global view. **(A)** Chromosome karyotype. **(B)** GC content. **(C)** Gene density. **(D)** DNA transposable elements (TEs). **(E)** Schematic presentation of the major inter-chromosomal relationships in the *S. cusia* genome.

*Strobilanthes cusia* is a diploid organism with 16 chromosome pairs. We further refined the chromosomal-level assembly based on the generated 106.27 Gb Hi-C clean data. The genome was linked using the ALLHiC pipeline. The final reference assembly included chromosome-scale pseudomolecules, with 16 pseudomolecules >20 Mb in length and covering 98.95% of the 913.74 Mb initial genome (Figure 1 and Supplementary Table 4). The number of scaffolds was lower, with an average length of ~8-fold >MinION (0.64 Mb) (Table 1). We used HiC-Pro to examine the Hi-C data assembly quality, with ~98.37 Gb on the data mapping, accounting for 92.57%. Unique mapped paired-end reads had ~7.9 Gb and a lib valid rate of ~37.93% (Supplementary Table 5). Taken together, the genome assembly of *S. cusia* showed high integrity and precision. In addition, our contig assembly shared high collinearity with the corresponding genomes from the previous reports (Supplementary Figure 2). Together, these data suggest that the genome assembly of *S. cusia* in this study is more complete compared with that published and of higher quality for subsequent analysis.

### Gene Prediction and Annotation

We annotated genes encoding proteins using the MAKER pipeline based on the transcriptome, homology, and *ab initio* prediction. We predicted a total of 32,974 genes (Table 1). The average gene length and intron sequence size were 3,399 and 306.51 bp, respectively; we identified 6.01 exons with an average exon length of 310.24 bp per gene. Compared with the MinION version, these gene models had much the longer average gene, exon, and intron lengths (Table 1). We evaluated the protein-coding gene completeness using BUSCO (98.5%), revealing 93% single-copy genes and 5.5% duplicated genes, which were slightly higher than those identified by MinION (91.39%) (Table 1 and Supplementary Table 6).

We functionally annotated the predicted protein sequences using NR, Swiss-Prot, and Pfam (Table 2); 96.33%, 79.63%, and 82.25% of the genes were homologous, respectively. We used COG, GO, and KEGG for annotation, with 88.74% of genes having COG, 43.9% with GO term classification, and 43% mapping to known biological pathways. These results illustrate the high reliability of the predicted gene model.

**Table 2:** Annotation statistics for the *S. cusia* genome.

**Annotation statistics for the genome**	**Number**	**Percent (%)**
Total proteins	32974	100
NR	31765	96.33
Swiss-Prot	26256	79.63
Pfam	27121	82.25
COG	29260	88.74
GO	14475	43.9
KEGG	14178	43
In all databases	13232	40.13
In at least one database	31828	96.52

Transcription factors regulate gene expression. Herein, we predicted TFs in the *S. cusia* genome using iTAK and showed that of the 32,974 genes encoding 2,666 TFs (Supplementary Table 7) were divided into 93 families, and MYB, AP2/ERF-ERF, bHLH, FAR1, and C2H2 were the major TF types.

We used miRDP software to identify miRNA, tRNA, and rRNA genes in the *S. cusia* genome from the miRBase and predicted rRNAs and tRNAs using RNAmmer and tRNAscan-SE, respectively, which revealed 122 miRNAs, 4,034 tRNAs, and 3,403 rRNAs (Supplementary Table 8).

### Repeat Annotation and Centromere Identification

The *S. cusia* genome has a large number of repeated sequences contained in 675.66 Mb and accounting for 74.04% of the genome (Supplementary Table 9). There are 429.62-Mb-long terminal repeat (LTR) retrotransposons, accounting for 47.08%. Non-LTR retrotransposons, comprising of LINE and SINE, represented 8.38% and 0.5%, respectively, whereas 14.23% comprised another type of DNA transposon. We identified 28,229 tandem repeats, constituting 4.22% of the *S. cusia* genome. A similar approach was applied to the *O. thomaeum* genome to identify centromeric repeats; 245 putative centromeric fragments with an average length of 77.93 bp were detected on the *S. cusia* chromosomes (Supplementary Table 10). The base centromeric consensus size was 172 bp. Among them, there were 56 on Chr1, forming the largest distribution. We also found 29 putative telomeric fragments. All chromosomes were distributed except for the deletion of Chr14 and Chr15 (Supplementary Table 11). The syntenic relationship is presented in a CIRCOS plot showing the TE distribution, gene density, and GC content (Figure 1).

### Identification and Characterization of Specific Gene Families

**Figure 2:**
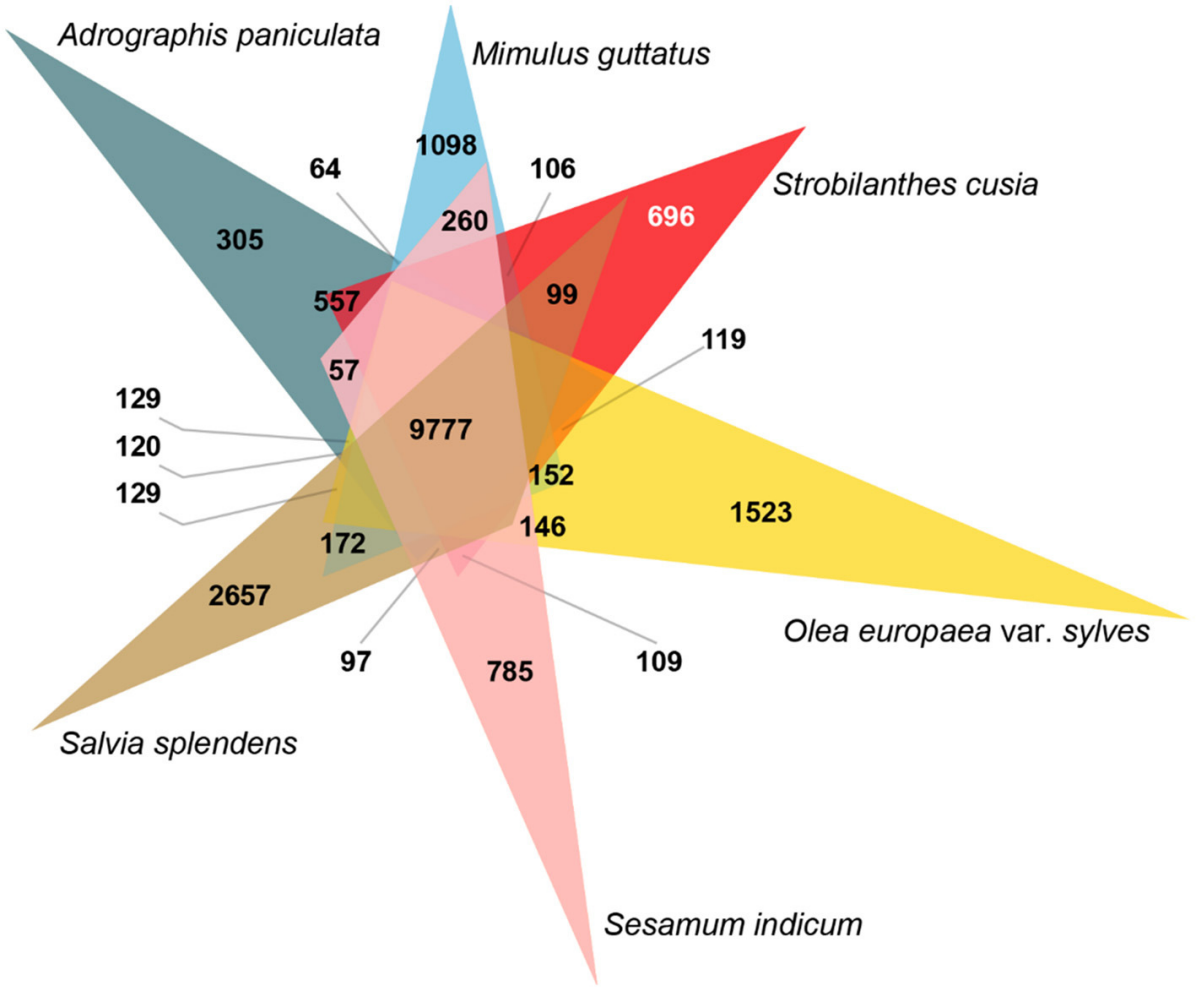
Analysis of Venn diagram of orthologous gene families. Six species (*S. cusia, Mimulus guttatus, Andrographis paniculata, Salvia splendens, Sesamum indicum*, and *Olea europaea* var. *sylvestris*) were exploited to generate the Venn diagram based on the gene family cluster analysis. The white number 696 in parentheses represents the gene family specific to *S. cusia* in all six plants considered.

Species-specific genes influence the adaptation of species to specific habitats; therefore, a comparative genomic analysis was conducted. Across the six Lamiales species genomes, 99,683 gene family clusters were observed among 231,423 genes (Supplementary Table 12). For the *S. cusia* genome, 32,974 genes were clustered into 16,955 gene families, 6,503 of which were single-copy genes and 696 were unique gene families compared with the other five closely related plants (Figure 2). GO enrichment analysis indicated that these specific gene families were mainly related to DNA integration, defense response, plant-type hypersensitive response, defense response to fungus, and regulation of salicylic acid biosynthetic process in the “biological process” term and cytochrome-c oxidase activity, and shikimate *O*-hydroxycinnamoyltransferase activity in the “molecular function” term (Supplementary Table 13). Notably, the unique gene families were exceedingly enriched in numerous plant defense functions and secondary metabolism. These enriched genes may contribute to the wide distribution and strong environmental adaptability of *S. cusia*.

### Evolution of the *S. cusia* Genome

**Figure 3:**
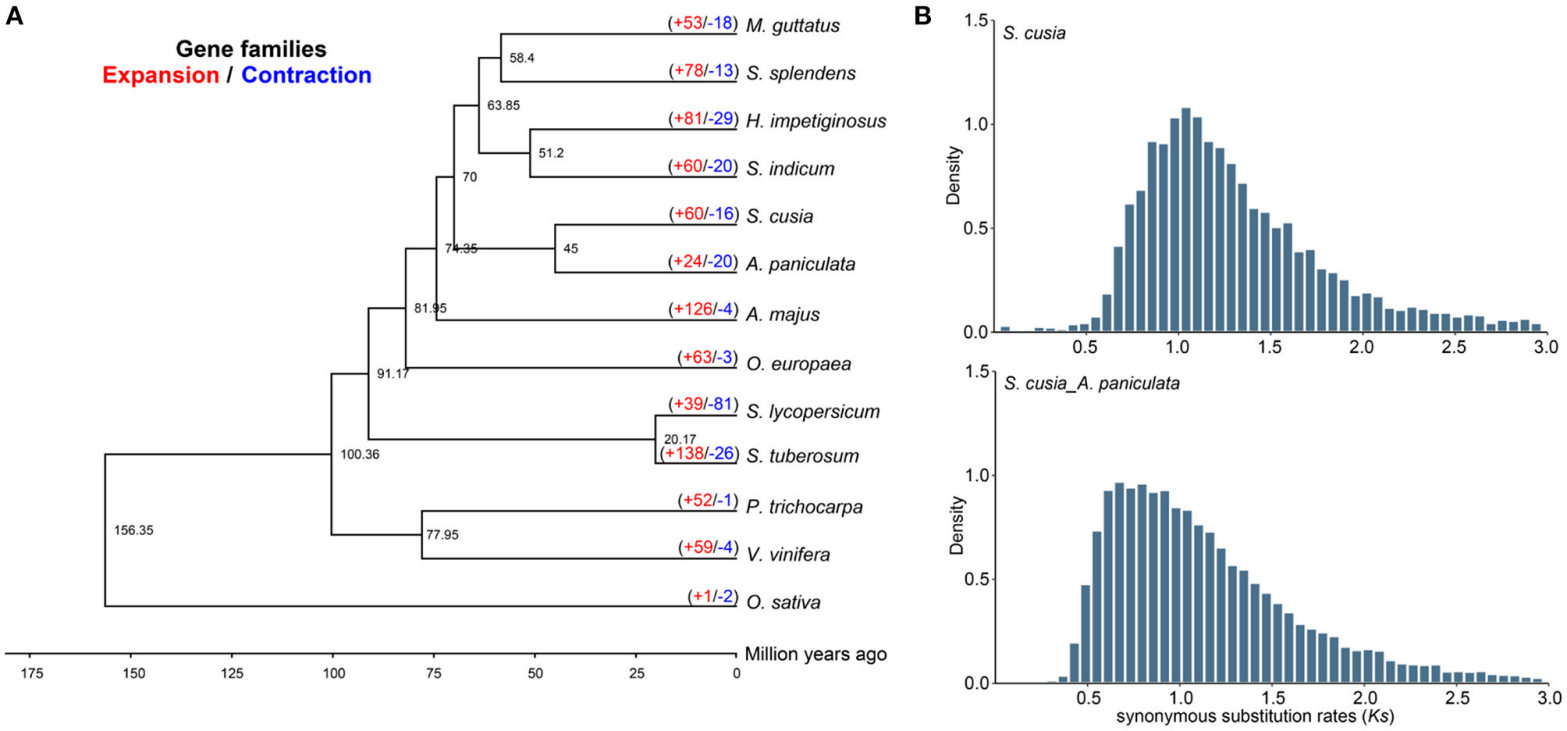
Comparative genomic and phylogenetic relationship analyses. **(A)** Phylogenetic relationship between *S. cusia* and other representative plant species. The divergence times among different plant species are labeled in the bottom. The red number on each branch represents expansion gene families, and blue number indicates the number of contracted gene families. **(B)** Density distribution of *Ks* values between syntenic genes of compared genomes.

We selected 65 single-copy genes among the 13 species to construct a phylogenetic tree, which showed that *S. cusia* was a sister to *A. paniculata*. Molecular dating using r8s with fossil calibration indicated that *S. cusia* and *A. paniculata* evolved ~45 Mya (Figure 3A). We further estimated the divergence time between these species using the density distribution of *Ks* (Figure 3B). The *Ks* values of the orthologues among species pairs revealed a peak of 0.65–0.85 for *S. cusia* and *A. paniculata*, with a corresponding divergence time of 50–65 Mya. Next, we used *Ks* between collinear paralogous genes to identify potential WGD events based on the assumption that the number of silent substitutions per site between two homologous sequences increases in a relatively linear manner over time. A density plot of *Ks* values for the collinear gene pairs suggested that *S. cusia* experienced an ancient polyploidization event with a peak value of ~1.157, and no specific WGD occurred in the *S. cusia* genome after divergence from Eudicots. The WGD data provide a foundation for genome evolution studies of *S. cusia*.

In *S. cusia*, 13,796 gene families were identified, among which 60 and 16 gene families showed rapid expansion and contraction (*P*-value), respectively (Figure 3A). Compared with closely related species of *A. paniculata* (24 expansion/20 contraction), *S. cusia* showed distinctly higher gene family expansion than contraction. In total, 60 expanded gene families were annotated to GO terms (Supplementary Table 14) and KEGG pathways (Supplementary Table 15). The GO analysis showed that the expanded orthogroups were related to spermidine hydroxycinnamate conjugate biosynthetic process, Penta cyclic triterpenoid metabolic process, Penta cyclic triterpenoid biosynthetic process, ethylene-activated signaling pathway, regulation of transcription, DNA-templated, and regulation of gene expression. The KEGG analysis showed that the most expanded genes were clustered in the MAPK signaling pathway, plant-pathogen interaction, diterpenoid biosynthesis, carotenoid biosynthesis, sesquiterpenoid and triterpenoid biosynthesis, and steroid biosynthesis. These expanded gene families were mainly concentrated in the pathways related to environmental adaptation and secondary metabolism, which are important in the biosynthesis of active ingredients and interaction between *S. cusia* and its growth environment.

### Transcriptome Analysis

**Figure 4:**
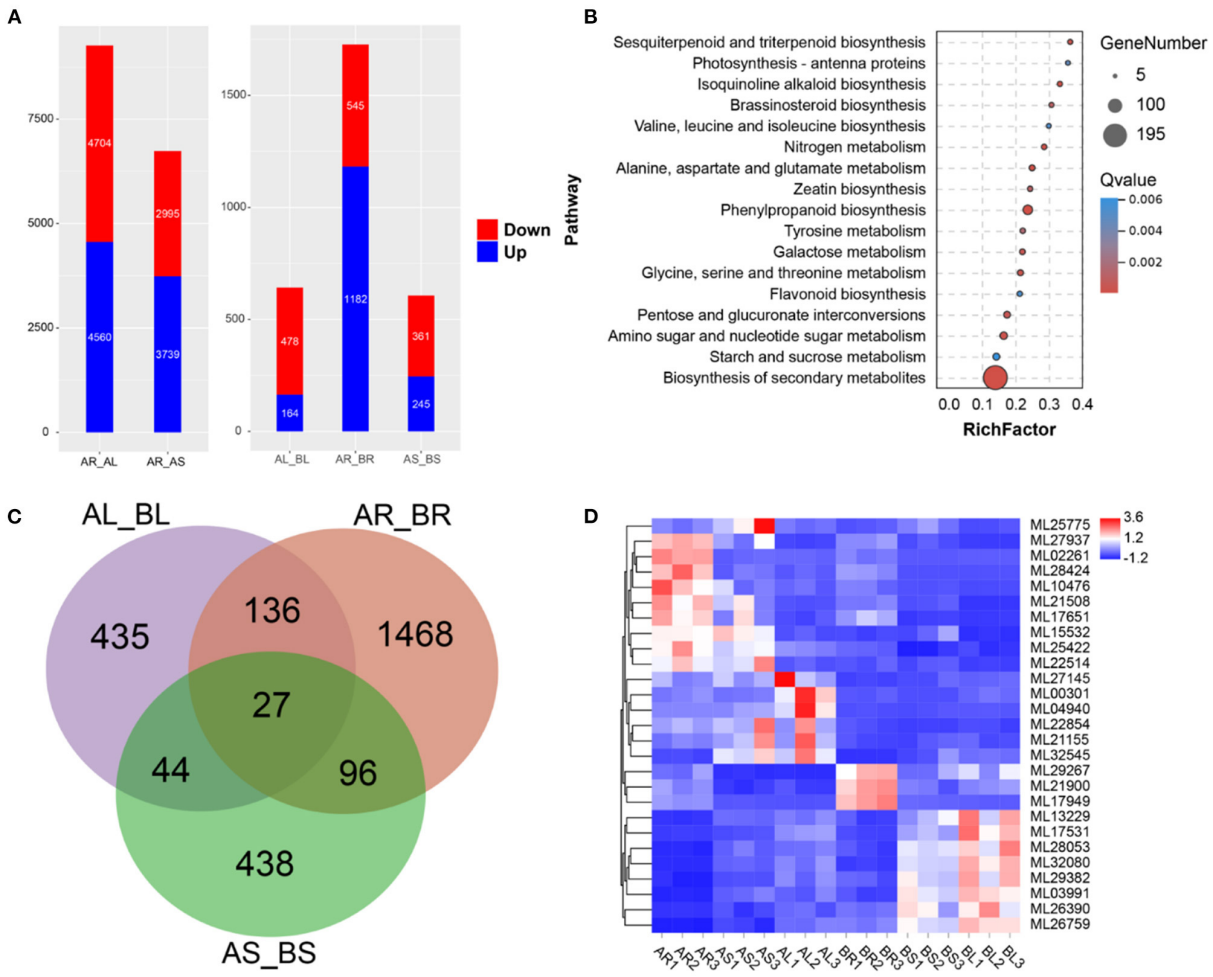
Transcriptome analysis. **(A)** Differentially expressed genes (DEGs) between the leaves, stems, and roots. AR_AL: DEGs in leaves relative to roots; AR_AS: DEGs in stems relative to roots; AL_BL: DEGs in leaves of treated sample relative to control sample. AR_BR: DEGs in roots of treated sample relative to control sample. AS_BS: DEGs in stems of treated sample relative to control sample; **(B)** Kyoto Encyclopedia of Genes and Genomes (KEGG) enrichment analysis of DEGs in treated samples relative to control samples. **(C)** Venn diagram of DEGs; **(D)** Expression of 27 DEGs in the leaves, stems, and roots.

Expression analysis was conducted using transcriptome data and *S. cusia*-annotated gene information. In the control group, 9,264 DEGs (upregulated: 4,704 and downregulated: 4,560) and 6,734 DEGs (upregulated: 2,995 and downregulated: 3,739) were in the leaves and stems, respectively, relative to the roots (Figure 4A). Following MeJA treatment, 2,975 DEGs were significantly differentially expressed in 32,974 annotated genes, of which 478 DEGs were upregulated and 164 DEGs were downregulated in MeJA-treated leaves, 545 DEGs were upregulated and 1,182 DEGs were downregulated in MeJA-treated roots, and 361 DEGs were upregulated and 245 DEGs were downregulated in MeJA-treated stems (Figure 4A). DEGs in the roots were quite different from those in the stems and leaves, which may be related to tissue specificity. These results indicated that the roots were the most sensitive to MeJA treatment, and the stems were the least sensitive. Through comparison of the data with the KEGG database, the metabolic pathway of *S. cusia* affected by MeJA induction was explored. KEGG enrichment analysis showed that 2,975 DEGs were clustered in the biosynthesis pathways of secondary metabolites, phenylpropanoid biosynthesis, flavonoid biosynthesis, alanine, aspartate and glutamate metabolism, pentose and glucoronate interconversions, sesquiterpenoid, triterpenoid biosynthesis, galactose metabolism, and photosynthesis-antenna proteins (Figure 4B). Among them, we identified 27 DEGs in the roots, stems, and leaves compared with the control group (Figures 4C,D). These annotation pathways and significant DEGs provide valuable information for studying the molecular regulatory mechanism of MeJA treatment for effectively enhancing the accumulation of IAs in *S. cusia*.

### Identification, Characterization, and Expression Analysis of LSGs

**Figure 5:**
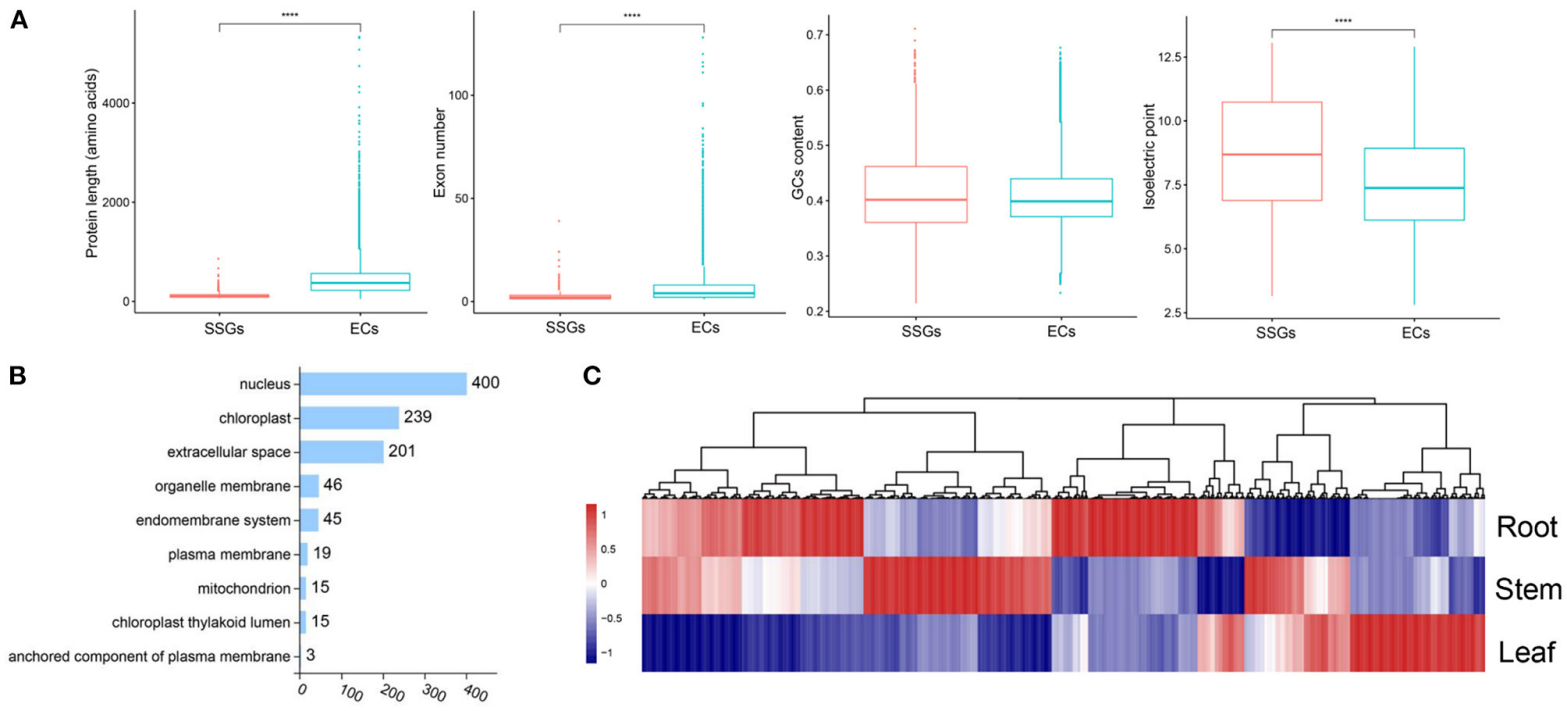
Analysis of lineage-specific genes (LSGs) in *S. cusia*. **(A)** Box-plot comparisons of protein length, exon number, GC content, and isoelectric point for the *Strobilanthes*-specific genes (SSGs) and evolutionarily conserved genes (ECs) in the *S. cusia*. SSGs mean the *S. cusia* LSGs; ECs mean the *S. cusia* evolutionary conserved genes. **(B)** Number of SSGs assigned to different subcellular locations. **(C)** Expression pattern of SSGs in the roots, stems, and leaves at three developmental stages.

The production of LSGs promotes the evolution and morphological diversity of species (Ma et al., 2020). Some research suggested that the biological functions of LSGs are related to the unique biological characteristics and environmental adaptability of the species. Based on the previous methods for LSG identification (Chen et al., 2020), 983 *Strobilanthes*-specific genes (SSGs) were identified, comprising 2.98% of the entire genome (Supplementary Table 16). In total, 31,992 ECs were identified. Gene duplication is the most important mechanism of LSG origin. In this study, we identified 195 SSGs originating from gene duplication, accounting for 19.86% of the SSGs (Supplementary Table 16). We compared the structural features of SSGs and ECs; the average EC length (442 aa) was significantly > that of the SSGs (112 aa) by ~3.65-fold, which was attributed to the presence of fewer exons in SSGs (Figure 5A). We also compared the isoelectric point of SSGs (8.64), which was significantly higher than that of ECs (7.54) (Figure 5A), and analyzed the SSG distribution on chromosomes based on the annotation information (Supplementary Table 16). In total, 980 SSGs were distributed on 16 chromosomes. Chr2 and Chr4 showed the largest number of SSGs. Subcellular localization is an important factor in protein function research and understanding the subcellular localization of proteins can help to determine their biological functions. The 840 SSGs were primarily localized in the nucleus, chloroplast, and extracellular space (400, 239, and 201, respectively), accounting for ~85.45% of all SSGs (Figure 5B). The analyses of the expression patterns of SSGs were conducted using RNA-seq data. Most SSGs showed tissue expression specificity (Figure 5C). These LSGs are valuable genetic resources for studying *Strobilanthes*-specific traits; how they participate in the complex biological network and their roles in a very short evolutionary time require further analysis.

### Identification of bHLHx Family

**Figure 6:**
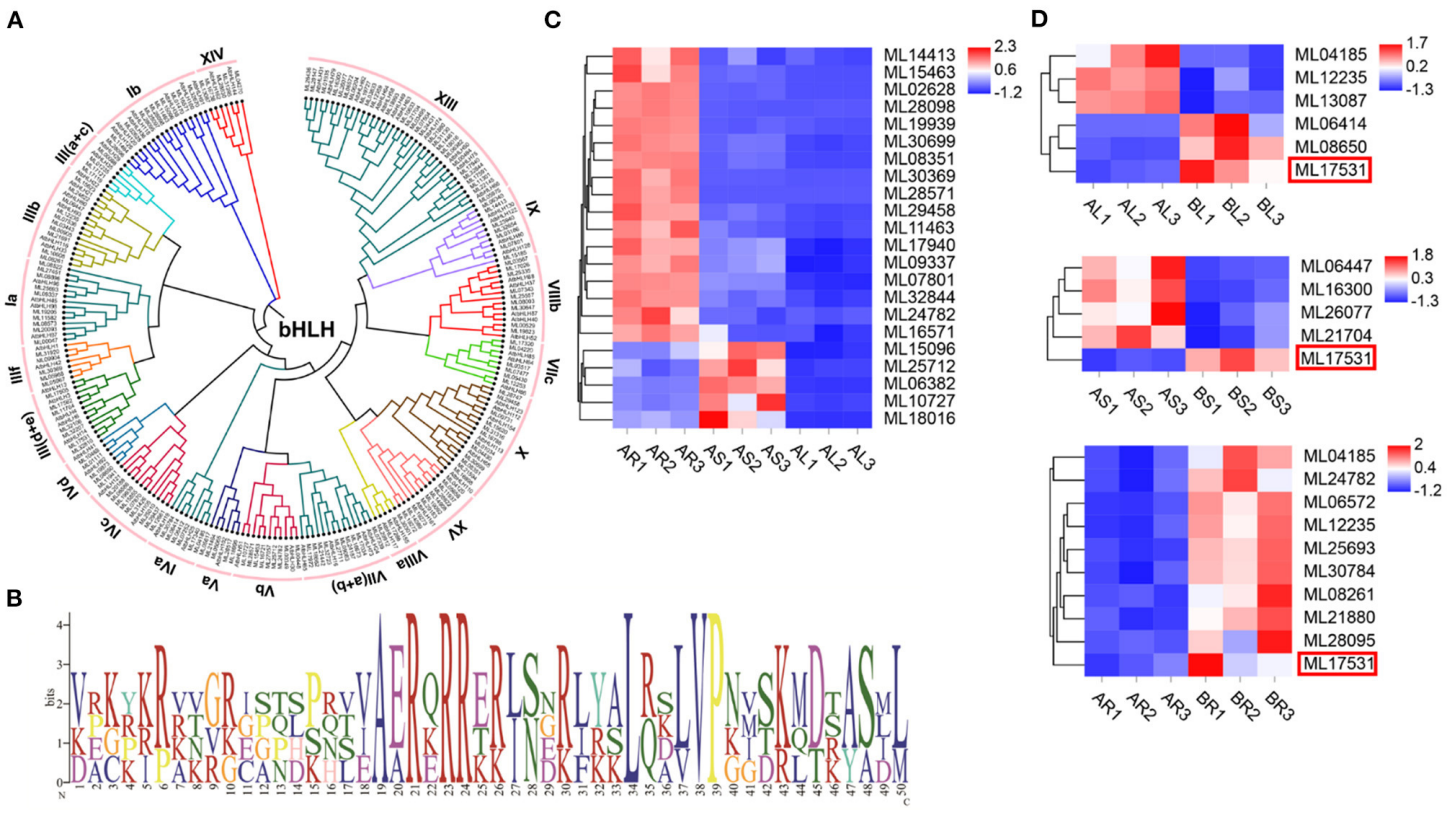
The basic helix-loop-helix (bHLH) in *S. cusia* genome. **(A)** Phylogenetic tree of bHLH based on the protein sequence alignments from *S. cusia* and *Arabidopsis* using MEGA X with the neighbor-joining methods. **(B)** Logo showed the conserved amino acid residues within motif. **(C)** *bHLH* gene members with significantly up and downregulated expression in leaf and stem relative to root. **(D)** *bHLH* gene members with significantly upregulated and downregulated expression in leaf and stem relative to control sample.

Among the 93 *S. cusia* TFs identified, bHLH is one of the largest families. In total, 173 bHLH family members were identified in the *S. cusia* genome (Supplementary Table 17). ML16673 encodes the highest molecular weight bHLH protein in the bHLH family at 717 amino acids (136,757.33 kDa). In comparison, ML19273, ML14908, and ML19092 encode the bHLH protein with the smallest molecular weight consisting of 93 amino acids (34,371.39 kDa). The isoelectric points of the proteins within the bHLH family recorded in *S. cusia* ranged from 4.5 for ML00529 to 10.78 for ML31426. A phylogenetic tree of the 173 bHLHs in *S. cusia* was constructed using MEGA7 software, and they were classified based on the taxonomy of the subfamily *Arabidopsis*. The results showed that the 173 bHLHs could be classified into 20 subfamilies, with the most distributed gene being subfamily XIII (Figure 6A). The gene family analysis showed that the members of bHLH had undergone significant expansion, whereas analysis of the bHLH amino acid conserved motifs using MEME showed that the N-terminal basic region contains the highly conserved H-E-R sequence (His-Glu-Arg), which is essential for E-box recognition and binding upstream of the target gene promoter. In the C-terminal HLH region, positions 34 and 50 Leu are highly conserved as positions 37 and 38 Leu/Val; these amino acid residues are important for dimer formation and function (Figure 6B). We also analyzed the expression of the 173 bHLHs, 17 of which were significantly downregulated in the stems and leaves compared with those in the roots, and five were significantly upregulated in the stems and significantly downregulated in the leaves compared with those in the roots (Figure 6C). In the transcriptome data following MeJA treatment, three *bHLH* genes were significantly upregulated and downregulated in the leaves, respectively, compared with those in the control group. In the stem, four genes were significantly downregulated, and one was significantly upregulated, whereas 10 genes were significantly upregulated in the root, with ML17531 significantly upregulated in the roots, stems, and leaves compared with those in the control group (Figure 6D).

### Genes Involved in the IA Biosynthesis Pathway

**Figure 7:**
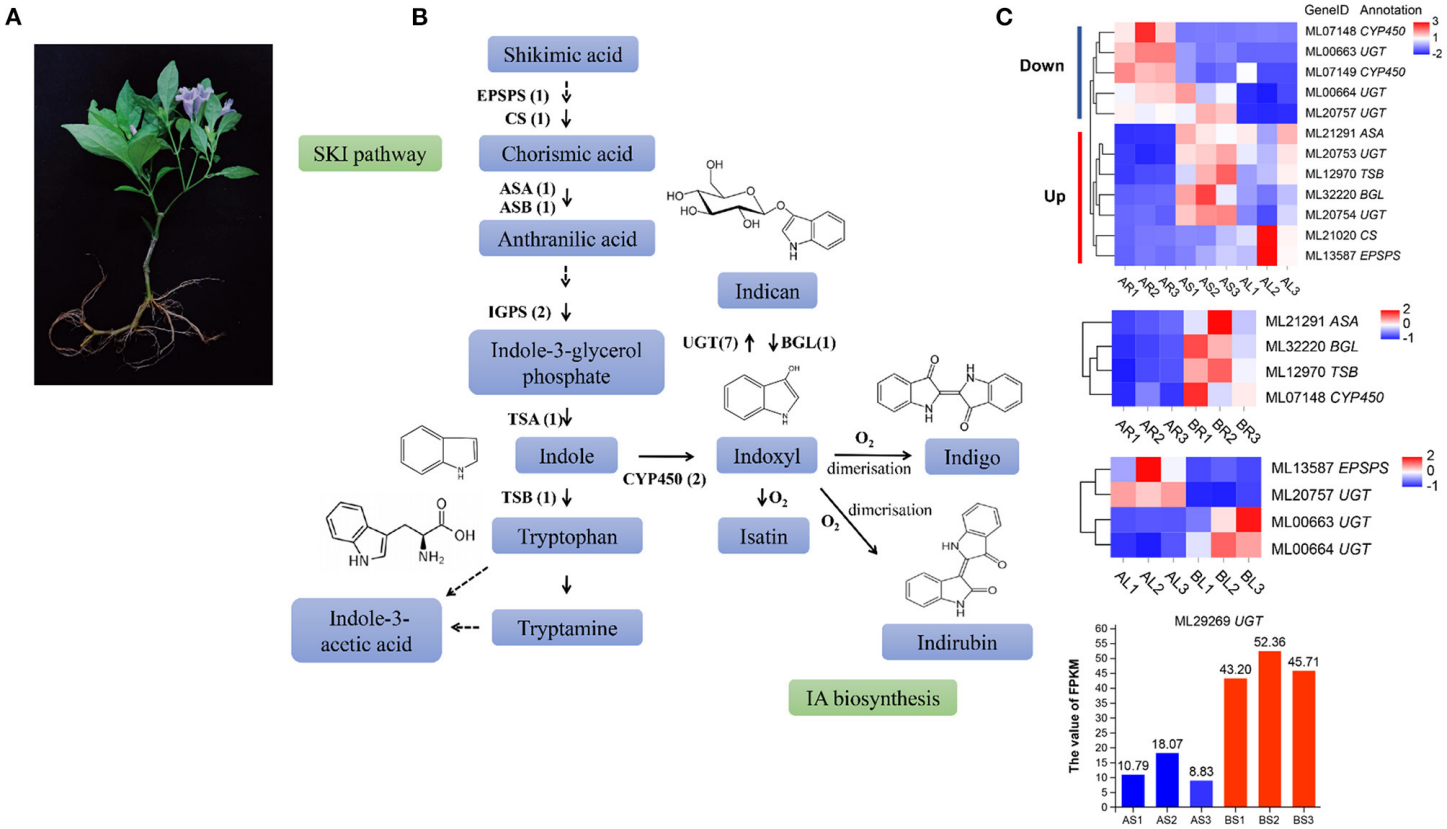
Analysis of indole alkaloid (IA) biosynthesis in *S. cusia*. **(A)** *S. cusia* whole plant display. **(B)** Putative biosynthesis pathway of IAs in *S. cusia*. **(C)** Heat map showing the expression pattern of genes associated with IA biosynthesis in different tissues including the roots, stems, and leaves.

*Strobilanthes cusia* is a natural antibacterial and antiviral raw material or Chinese herbal medicine, and the monomer components or mixtures in its roots, stems, and leaves exhibit significant therapeutic effects. These active substances are mainly derived from the IA biosynthesis pathway (Figure 7A). We further evaluated 10 key enzymes associated with the IA synthesis pathway for homology searching and confirmed 18 IA-related coding genes, such as, seven copies of UDP-glucuronosyltransferase (*UGT*), two of indole-3-glycerol phosphate synthase (*IGPS*), two of cytochrome P450 monooxygenase (*CPY450*), *EPSPS*, chorismate synthase (*CS*), anthranilate synthase a-subunit (ASA), anthranilate synthase β-subunit *(ASB)*, β-glucosidase *(BGL)*, tryptophan synthase α-subunit *(TSA)*, and tryptophan synthase β-subunit (*TSB*) in the assembled genome of *S. cusia* (Figure 7B). Compared with the stems and leaves, the expression of three *UGT* (ML00663/ML00664/ML20757) and two *CYP450* (ML07148/ML07149) genes in the roots decreased significantly, whereas the expression of two *UGT* (ML20753/ML20754), one *ASA* (ML21291), one *TSB* (ML12970), one *BGL* (ML32220), one *CS* (ML21020), and one *EPSPS* (ML13587) gene increased significantly. The expression levels of four genes (ML21291/ML32220/ML12970/ML07148) were greatly increased in the roots treated with MeJA. Two genes (ML13587/ML20757) were significantly downregulated and two *UGT* genes (ML00663/ML00664) were significantly upregulated in MeJA-treated leaves. One *UGT* gene (ML29269) was significantly upregulated in MeJA-treated stems (Figure 7C). These DEGs may be closely related to the synthesis of IAs, such as, indigo and indirubin and can provide valuable information on the metabolic regulation of the active ingredients in *S. cusia*.

## Discussion

*Strobilanthes cusia* is a characteristic medicinal plant with a typical metabolic pathway of IAs. The market demand is very high for this Chinese medicine with great development potential. Owing to the low content of natural products in the plants, extraction steps are cumbersome, and the yield is low, making it necessary to obtain sufficient medicinal natural products using new approaches. It is particularly important to analyze the genetic background and secondary metabolic pathways of medicinal plants. In this study, we present a high-quality reference genome assembly of *S. cusia* determined using Illumina, PacBio CCS, and Hi-C sequencing data. The resulting total genome size was 913.74 Mb (the estimated size was 826.37 Mb for MinION versions based on k-mer composition) (Xu et al., 2020). A remarkable increase in the size of the contig and scaffold N50 was achieved using the new “PacBio CCS” assembly; particularly, the contig N50 was 8-fold larger than the previously reported (Table 1). The assembly precision based on the Hi-C contact map was improved compared with that of the previous assemblies. These data provide an important reference for improving the assembly quality of the current medicinal plant genome. The content of pharmacodynamics components in Chinese herbal medicine is typically related to various factors, such as, the variety, place of origin, medicinal parts, harvest season, growth period, and other factors. Our assembled genetic information provides a reliable molecular basis for explaining these differences. After enrichment analysis of species-specific genes and expansion gene families, we found that the gene families were concentrated in pathways involved in the plant defense response and secondary metabolism. This phenomenon is related to the secondary metabolites for adaptation to the living environment and is consistent with the substances produced by resisting the negative conditions of the natural world. Additionally, we used comparative genomics to identify LSGs in the *S. cusia* genome and subsequently analyzed their origin, sequence structure, and expression patterns. As LSGs represent important drivers for the generation of new functions and phenotypic changes in species, the LSGs identified in the current study are thought to be important for *S. cusia* breeding.

Currently, the synthesis pathway of IAs, a key medicinal ingredient in *S. cusia*, has been primarily derived based on the presumption of microbial metabolism and the studies on other indigo source plants (Jin et al., 2016; Ma et al., 2016; Kang et al., 2020). It is generally accepted that the synthesis of indigo, indirubin, and indole glycosides in *S. cusia* involves the shikimate and indole pathways. Regulation of the biosynthetic pathway of target secondary metabolites through exogenous elicitors is regarded as an important method for greatly increasing the metabolite content. High-performance liquid chromatography (HPLC) showed that the content of indigo in the leaves and roots of *S. cusia* was significantly increased after treatment with exogenous MeJA (Lin et al., 2019). MeJA is thought to activate or inhibit the activity of corresponding TFs through signal transduction, thereby regulating the expression of key enzyme genes in the secondary metabolic pathway and ultimately affecting the synthesis of secondary metabolites (Wang et al., 2019; Zhou et al., 2021). Therefore, we performed a homology search, high-quality genome functional annotation, and transcriptome analysis of *S. cusia* before and after MeJA treatment to identify IA-related coding genes to better understand the biological characteristics of *S. cusia* and *in vivo* biosynthesis pathways for indigo, indirubin, and indole glycosides. We identified one *EPSPS* and one *CS*, which encode two enzymes responsible for catalyzing the chorismic acid synthesis from precursor substances, as well as key enzyme genes in the shikimate pathway (Wang et al., 2014; Yu et al., 2019). One *ASA* gene and one *ASB* gene were involved in catalyzing the synthesis of anthranilic acid from chorismic acid, whereas ASA is considered to have a rate-limiting effect on indigo synthesis. We also identified two *IGPS* and two *TSA* genes in the *S. cusia* genome. Anthranilic acid synthesizes indole under the combined action of IGPS and TSA. ^13^C-NMR and mass spectrometry revealed that indole serves as the precursor of indoxyl derivatives, rather than l-tryptophan in plants (Xia and Zenk, 1992). Our identification of genes related to the shikimate pathway in the *S. cusia* genome supports that indole heterocycles in the backbone structures of indigo and indirubin are derived from the shikimate pathway. Additionally, two *CYP450* and seven *UGT* genes were identified: CYP450 oxidizes indole to indoxyl, which is then glucosylated by UGT to form indoxyl-3-*O*-β-d-glucoside (indican) (Marcinek et al., 2000). One *BGL* gene was identified. When exposed to external influences or leaf senescence, indican was reversibly hydrolyzed under BGL to decompose into indoxyl. Subsequently, indigo and indirubin can be synthesized by the polymerization of indophenol under aerobic conditions. However, the functionalities of the obtained candidate genes require further validation *in vivo*. Analyzing the genome of *S. cusia* after MeJA treatment to obtaining candidate genes related to the synthesis of IAs provides valuable data resources for studying the medicinal properties of *S. cusia*.

In addition to enzyme genes, TFs are thought to play an important role in secondary metabolism by activating or inhibiting gene transcription through binding with *cis*-acting DNA elements. In recent years, more and more bHLH TFs have been addressed to be closely related to the alkaloid metabolism in medicinal plants such as, *Catharanthus roseus, Taxus cuspidata*, and *Coptis japonica* (Yamada et al., 2011; Lenka et al., 2015; Patra et al., 2018). MeJA induction can significantly increase the content of indigo in *S. cusia* (Lin et al., 2019), and bHLHs, especially MYC proteins, can play a key role in the signal transduction process after JA perception (Fernández-Calvo et al., 2011). To discover the candidate bHLHs involved in JA-mediated regulation of secondary metabolism in *S. cusia*. Based on the sequence similarity, evolutionary relationships, and motif diversity, 173 bHLH proteins were identified and characterized. Phylogenetic analysis and the domain distribution of bHLH reflected the conservatism of the functional structure of the family (Li et al., 2006; Carretero-Paulet et al., 2010; Pires and Dolan, 2010). The tissue-specific expression profiles of the bHLH TF family in different tissues (roots, stems, and leaves) were analyzed using RNA-seq data. The expression patterns of *bHLH* differed significantly among the various tissues; however, most *bHLH* levels in the stems and leaves were significantly lower than those in the roots. Moreover, compared with that in the control group, ML17531 was significantly upregulated in the roots, stems, and leaves following MeJA treatment, suggesting that this transcription factor is involved in synthesizing IAs in *S. cusia*. Our research identified and supplemented candidate enzyme genes and TFs involved in the biosynthetic pathways of indigo, indirubin, and indole glycosides in *S. cusia*. These results provide a selectable target for the regulation of IA metabolic flow and valuable information for cultivating *S. cusia* varieties with high medicinal ingredient contents through metabolic engineering.

## Conclusion

We propose a high-quality genome for the medicinal plant *S. cusia*, which we sequenced using the PacBio CCS sequencing platform, with an assembled genome size of ~913.74 Mb. From the assembled genomes, we identified 675.66 Mb of repetitive sequences were identified, representing 74.04% of the genome. The chromosome-level genome, resulting from Hi-C data, yielded a contig N50 size of 35.59 Mb and scaffold N50 size of 68.44 Mb. We also predicted 32,974 protein-coding genes, of which 96.52% had annotations in public databases. Further, we identified 245 putative centromeric fragments, 29 putative telomeric fragments, and 983 SSGs in *S. cusia*. We identified the key enzyme genes and candidate bHLH family TFs associated with the IA pathway in *S. cusia*. In conclusion, high-quality genome sequencing of *S. cusia* provided insight into the systematic study of IA synthesis in this medicinally and economically important Chinese herbal medicine. Our study provides a theoretical basis for the breeding of *S. cusia* varieties and the production of IAs through new methods such as, synthetic biology.

## Data Availability Statement

The original contributions presented in the study are publicly available. This data can be found here: the data that support the findings of this study have been deposited into CNGB Sequence Archive of CNGBdb with accession number CNP0001632 (https://db.cngb.org/). The genome assembly, annotation (gff), protein, coding sequences (CDS) and cDNA sequences can be found under assembly accession number CNA0019301.

## Author Contributions

YH, DM, and DW designed and coordinated the entire project. YH and DM led, performed the entire project together, performed the data analysis, and wrote the manuscript. DW supervised the project. GC and XM collected the samples for sequencing. XZ and XQ submitted data to the database. SN, QY, QD, and PL participated in manuscript revision. All authors read and approved the final manuscript.

## Funding

This research was supported by the National Natural Science Foundation of China (No. 81573517), Natural Science Foundation of Fujian Province (No. 2019J01827), Science and Technology Innovation Project of Fujian Agriculture and Forestry University (No. CXZX2020011A), and the Central Special Project for Fujian Local Science and Technology Development (No. 2020L3025).

## Conflict of Interest

The authors declare that the research was conducted in the absence of any commercial or financial relationships that could be construed as a potential conflict of interest.

## Publisher's Note

All claims expressed in this article are solely those of the authors and do not necessarily represent those of their affiliated organizations, or those of the publisher, the editors and the reviewers. Any product that may be evaluated in this article, or claim that may be made by its manufacturer, is not guaranteed or endorsed by the publisher.
