# Supplementary material for: High-Quality Genome of the Medicinal Plant Strobilanthes cusia Provides Insights Into the Biosynthesis of Indole Alkaloids
Source: Front Plant Sci. 2021 Sep 30;12:742420. doi: 10.3389/fpls.2021.742420 (PMC8515051; doi:10.3389/fpls.2021.742420)
Supplement: Supplementary file 1 [file Data_Sheet_1.docx]

***Supplementary Material***


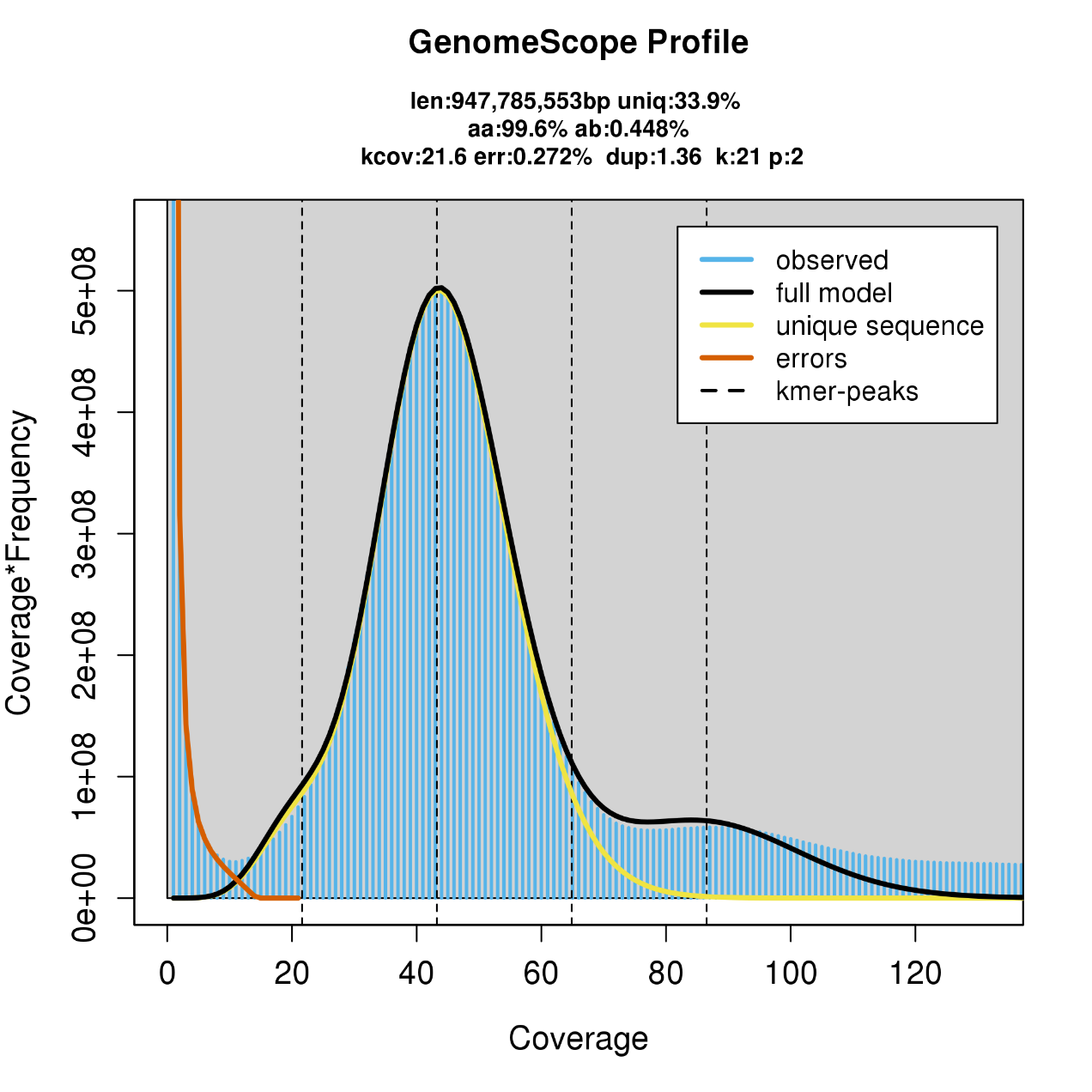


**Supplementary Figure 1. Assessment of genome size and complexity based on K‐mers methods.**

**
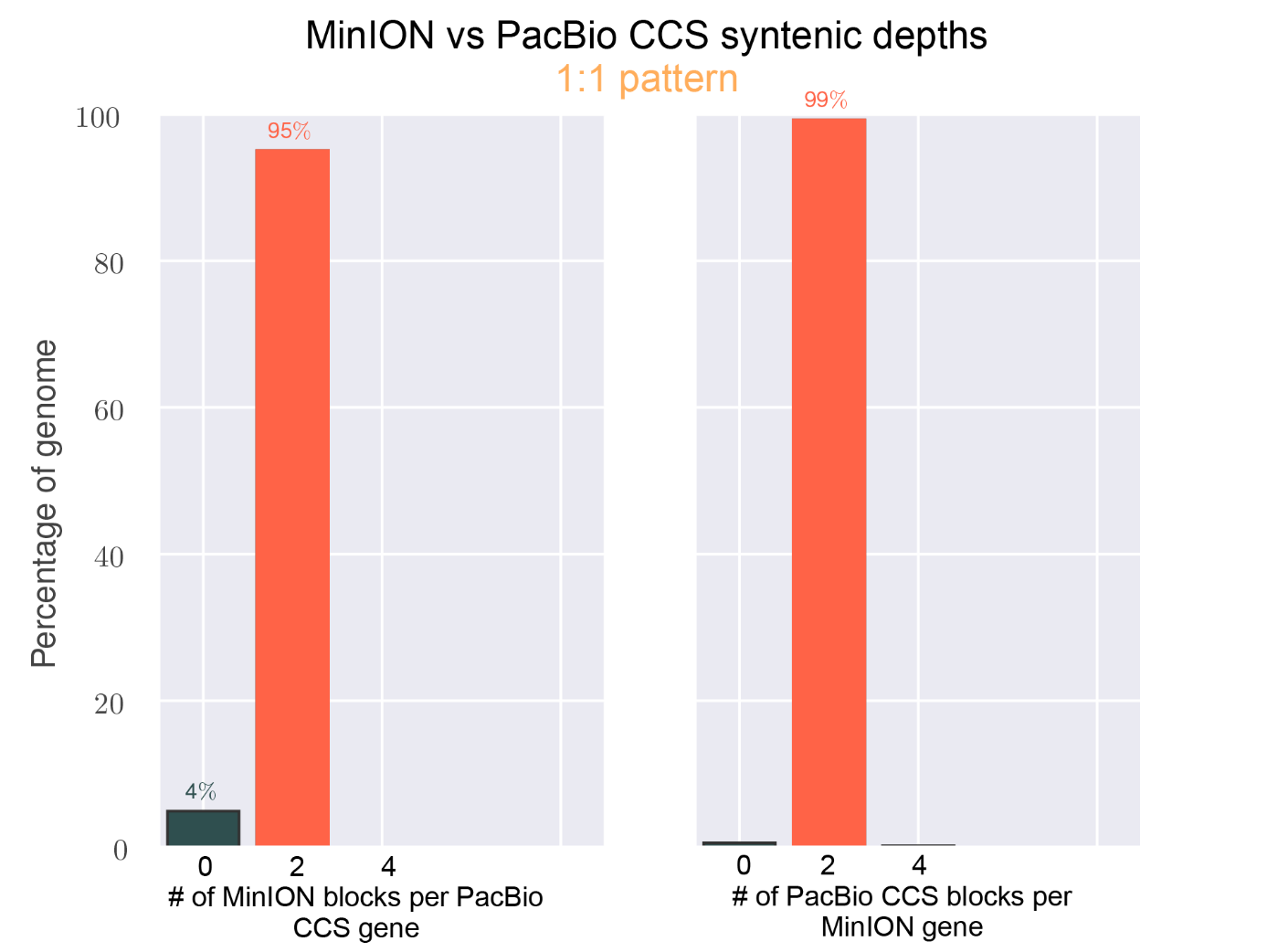
**

**Supplementary Figure 2.** **Collinear analysis of the assemblies of *S. cusia* genome**

# Supplementary Table 1. Sequencing information of the *S. cusia*.

| **Items** | **Illumina** | **PacBio CCS** |
| --- | --- | --- |
| Total Number of reads | 667,375,156 | 37,826,723 |
| Total Number of sequenced Bases (Gb) | 49.98 | 483.97 |
| Mean reads length (bp) | 150 | 12,668 |
| N50 (bp) | 150 | 13,970 |
| Coverage (×) | 52.73 | 531.73 |

# Supplementary Table 2. BUSCO analysis of genome assembly.

| **Description** | **Number** | **Percentage (%)** |
| --- | --- | --- |
| Complete BUSCOs (C) | 1344 | 97.8 |
| Complete and single-copy BUSCOs (S) | 1269 | 92.3 |
| Complete and duplicated BUSCOs (D) | 75 | 5.5 |
| Fragmented BUSCOs (F) | 8 | 0.6 |
| Missing BUSCOs (M) | 23 | 1.6 |
| Total BUSCO groups searched | 1,375 | 100 |

# Supplementary Table 3. Assessment of genome consistency based on Illumina reads.

| **Items** | **Statistics** |
| --- | --- |
| Number of reads | 313,197,292 |
| Data size (Gb) | 49.98 |
| Mapped bases (Gb) | 49.91 |
| Mapping rate (%) | 99.85 |
| Genome Length (Gb) | 913.74 |
| Mean Depth | 48.13 |
| Coverage Rate (%) | 98.58 |

# Supplementary Table 4. Pseudomolecule length statistics after Hi-C assisted assembly.

| **Pseudomolecule** | **Length (bp)** |
| --- | --- |
| Chr1 | 94,747,430 |
| Chr2 | 87,398,577 |
| Chr3 | 77,968,717 |
| Chr4 | 72,211,168 |
| Chr5 | 72,205,009 |
| Chr6 | 68,439,447 |
| Chr7 | 66,544,764 |
| Chr8 | 64,376,749 |
| Chr9 | 52,549,036 |
| Chr10 | 50,440,415 |
| Chr11 | 42,103,120 |
| Chr12 | 38,095,908 |
| Chr13 | 35,037,375 |
| Chr14 | 31,850,365 |
| Chr15 | 27,403,129 |
| Chr16 | 22,812,488 |

# Supplementary Table 5. Statistics of Hi-C mapping of the *S. cusia*.

| **Items** | **Statistics of mapping** |
| --- | --- |
| Clean Paired-end Reads | 354,791,653 |
| Unmapped Paired-end Reads | 26,372,724 |
| Unmapped Paired-end Reads Rate (%) | 7.433 |
| Paired-end Reads with Singleton | 227,855,881 |
| Paired-end Reads with Singleton Rate (%) | 64.222 |
| Multi Mapped Paired-end Reads | 31,464,146 |
| Multi Mapped Ratio (%) | 8.868 |
| Unique Mapped Paired-end Reads | 69,098,902 |
| Unique Mapped Ratio (%) | 19.476 |
|  | **Statistics of valid reads** |
| Unique Mapped Paired-end Reads | 69,098,902 |
| Dangling End Paired-end Reads | 25,227,002 |
| Dangling End Rate (%) | 36.509 |
| Self Circle Paired-end Reads | 534,030 |
| Self Circle Rate (%) | 0.773 |
| Dumped Paired-end Reads | 7,648,870 |
| Dumped Rate (%) | 11.069 |
| Interaction Paired-end Reads | 34,807,345 |
| Interaction Rate (%) | 50.373 |
| Lib Valid Paired-end Reads | 26,211,863 |
| Lib Valid Rate (%) | 37.934 |
| Lib Dup (%) | 24.694 |

# Supplementary Table 6. BUSCO analysis of annotation completeness.

| **Description** | **Number** | **Percentage (%)** |
| --- | --- | --- |
| Complete BUSCOs (C) | 1355 | 98.5 |
| Complete and single-copy BUSCOs (S) | 1279 | 93.0 |
| Complete and duplicated BUSCOs (D) | 76 | 5.5 |
| Fragmented BUSCOs (F) | 6 | 0.4 |
| Missing BUSCOs (M) | 14 | 1.1 |
| Total BUSCO groups searched | 1,375 | 100 |

# Supplementary Table 7. Statistics transcription factors of the *S. cusia* genome.

| **Classify** | **Count** | **Classify** | **Count** | **Classify** | **Count** |
| --- | --- | --- | --- | --- | --- |
| MYB | 177 | AP2/ERF-AP2 | 27 | E2F-DP | 8 |
| AP2/ERF-ERF | 171 | zf-HD | 23 | CPP | 8 |
| bHLH | 171 | HB-BELL | 22 | AP2/ERF-RAV | 7 |
| FAR1 | 154 | SBP | 20 | Pseudo | 7 |
| C2H2 | 137 | NF-YB | 19 | BBR-BPC | 7 |
| NAC | 105 | Tify | 19 | DDT | 7 |
| WRKY | 98 | HB-other | 19 | EIL | 7 |
| bZIP | 78 | RWP-RK | 18 | CSD | 7 |
| Others | 76 | GARP-ARR-B | 17 | DBB | 6 |
| MYB-related | 73 | TRAF | 16 | LUG | 5 |
| C3H | 70 | GRF | 16 | C2C2-LSD | 4 |
| HB-HD-ZIP | 57 | Jumonji | 15 | DBP | 4 |
| GARP-G2-like | 54 | SWI/SNF-BAF60b | 15 | SWI/SNF-SWI3 | 4 |
| GRAS | 54 | HB-WOX | 14 | Coactivator | 3 |
| MADS-M-type | 52 | HB-KNOX | 13 | Rcd1-like | 3 |
| B3 | 52 | C2C2-CO-like | 12 | VOZ | 3 |
| LOB | 47 | HMG | 12 | Whirly | 3 |
| MADS-MIKC | 46 | PLATZ | 12 | MBF1 | 3 |
| PHD | 42 | IWS1 | 12 | SOH1 | 2 |
| C2C2-Dof | 40 | LIM | 11 | HB-PHD | 2 |
| SET | 39 | NF-YC | 11 | NF-X1 | 2 |
| Trihelix | 39 | TUB | 11 | RB | 2 |
| SNF2 | 39 | NF-YA | 10 | MED7 | 1 |
| mTERF | 39 | Alfin-like | 10 | ULT | 1 |
| HSF | 38 | ARID | 9 | HRT | 1 |
| TCP | 34 | C2C2-YABBY | 9 | LFY | 1 |
| GNAT | 34 | GeBP | 9 | S1Fa-like | 1 |
| B3-ARF | 32 | BES1 | 9 | SAP | 1 |
| C2C2-GATA | 32 | SRS | 9 | BSD | 1 |
| AUX/IAA | 30 | CAMTA | 9 | MED6 | 1 |
| OFP | 29 | TAZ | 8 | STAT | 1 |

# Supplementary Table 8. Statistical analysis of non-coding RNAs in the *S. cusia* genome.

| **Class** | **Number** | **Total length (bp)** | **Mean length (bp)** |
| --- | --- | --- | --- |
| miRNAs | 122 | 10991 | 90.09 |
| tRNAs | 4034 | 2948585 | 88 |
| rRNAs | 3403 | 10253043 | 3013 |

# Supplementary Table 9. TE annotation of the *S. cusia* genome.

| Type | Length (bp) | Genome coverage (%) |
| --- | --- | --- |
| DNA | 129842511 | 14.23 |
| SINE | 4554509 | 0.5 |
| LINE | 76433648 | 8.38 |
| LTR | 429617405 | 47.08 |
| Simple repeats | 9144270 | 1.00 |
| Low complexity | 1297514 | 0. 14 |
| Other | 469211 | 0.05 |
| Unclassified | 24298632 | 2.66 |
| Total length | 675657700 | 74.04 |

# Supplementary Table 10. The putative centromere regions on the *S. cusia* chromosomes.

| **seqid** | **start** | **end** | **period** | **copynum** | **consensusSize** | **pctmatch** | **pctindel** | **score** |
| --- | --- | --- | --- | --- | --- | --- | --- | --- |
| Chr01 | 29538 | 37536 | 172 | 46.3 | 172 | 83 | 1 | 6266 |
| Chr01 | 537191 | 653189 | 172 | 674.6 | 172 | 79 | 3 | 75024 |
| Chr01 | 653290 | 726454 | 172 | 424.3 | 172 | 69 | 7 | 25420 |
| Chr01 | 1310229 | 1415227 | 172 | 610.6 | 172 | 73 | 5 | 54786 |
| Chr01 | 37043825 | 37048632 | 172 | 27.9 | 172 | 85 | 1 | 3976 |
| Chr01 | 37053526 | 37069910 | 172 | 95.2 | 172 | 81 | 1 | 12718 |
| Chr01 | 37072193 | 37133989 | 172 | 357.9 | 172 | 64 | 8 | 26674 |
| Chr01 | 37148621 | 37152349 | 172 | 21.8 | 172 | 82 | 0 | 2988 |
| Chr01 | 37172045 | 37352968 | 172 | 1050.7 | 172 | 55 | 11 | 32378 |
| Chr01 | 59301560 | 59305494 | 172 | 23.1 | 172 | 69 | 5 | 1894 |
| Chr01 | 59306424 | 59313999 | 172 | 43.9 | 172 | 74 | 3 | 5037 |
| Chr01 | 61054625 | 61062403 | 172 | 45.7 | 172 | 76 | 5 | 4723 |
| Chr01 | 61138114 | 61162227 | 172 | 140.1 | 172 | 74 | 5 | 7896 |
| Chr01 | 61162223 | 61191944 | 172 | 173 | 172 | 73 | 5 | 15320 |
| Chr01 | 61196093 | 61204104 | 173 | 46.3 | 173 | 67 | 2 | 4891 |
| Chr01 | 61206258 | 61213791 | 173 | 43.5 | 173 | 69 | 1 | 5075 |
| Chr01 | 61231295 | 61249279 | 173 | 103.8 | 173 | 59 | 8 | 8087 |
| Chr01 | 61309088 | 61333294 | 173 | 139.8 | 173 | 53 | 12 | 6314 |
| Chr01 | 61362070 | 61364024 | 173 | 11.3 | 173 | 70 | 1 | 1365 |
| Chr01 | 61393262 | 61397114 | 173 | 22.3 | 172 | 70 | 3 | 2584 |
| Chr01 | 61429766 | 61444814 | 173 | 87.2 | 173 | 56 | 8 | 6215 |
| Chr01 | 61548571 | 61551521 | 173 | 17.1 | 173 | 70 | 3 | 2030 |
| Chr01 | 61733100 | 61737490 | 173 | 25.4 | 173 | 70 | 2 | 2973 |
| Chr01 | 61870722 | 61889677 | 173 | 109.6 | 173 | 56 | 7 | 8125 |
| Chr01 | 61886471 | 61933727 | 173 | 272.2 | 173 | 47 | 12 | 8889 |
| Chr01 | 62115177 | 62117216 | 173 | 11.8 | 174 | 67 | 3 | 1312 |
| Chr01 | 62159617 | 62552507 | 172 | 2281.5 | 172 | 66 | 8 | 85446 |
| Chr01 | 62566063 | 62639707 | 172 | 429.3 | 172 | 81 | 2 | 55601 |
| Chr01 | 62639702 | 62653818 | 172 | 82.2 | 172 | 86 | 0 | 11985 |
| Chr01 | 62653919 | 63153918 | 172 | 2904.4 | 172 | 66 | 8 | 140070 |
| Chr01 | 81416272 | 81453001 | 173 | 211.6 | 174 | 45 | 16 | 2427 |
| Chr01 | 81523472 | 81798327 | 172 | 1595.5 | 172 | 67 | 7 | 96145 |
| Chr01 | 81798326 | 81801207 | 172 | 16.9 | 172 | 85 | 1 | 2435 |
| Chr01 | 81801205 | 81809878 | 172 | 50.7 | 172 | 82 | 2 | 6533 |
| Chr01 | 81809873 | 82022326 | 172 | 1232.6 | 172 | 68 | 8 | 67148 |
| Chr01 | 82022314 | 82038612 | 172 | 94.8 | 172 | 85 | 0 | 13668 |
| Chr01 | 90504522 | 90527699 | 172 | 134.8 | 172 | 78 | 1 | 17091 |
| Chr01 | 90543123 | 90550945 | 172 | 45.6 | 172 | 82 | 0 | 6218 |
| Chr01 | 90618904 | 90626913 | 172 | 46.5 | 172 | 74 | 4 | 4380 |
| Chr01 | 90642462 | 91016727 | 172 | 2172.4 | 172 | 70 | 7 | 136593 |
| Chr01 | 91050543 | 91143764 | 173 | 536.6 | 173 | 47 | 13 | 19800 |
| Chr01 | 91164686 | 91185993 | 173 | 122.9 | 173 | 57 | 8 | 9132 |
| Chr01 | 91197081 | 91202745 | 173 | 32.7 | 173 | 68 | 1 | 3746 |
| Chr01 | 91203052 | 91536485 | 172 | 1939 | 172 | 79 | 3 | 183958 |
| Chr01 | 91536479 | 91549506 | 172 | 75.9 | 172 | 80 | 3 | 9181 |
| Chr01 | 91556496 | 91559005 | 172 | 14.6 | 172 | 86 | 1 | 2142 |
| Chr01 | 91559004 | 91640876 | 172 | 476.3 | 172 | 80 | 3 | 52058 |
| Chr01 | 92382976 | 92673159 | 172 | 1684.9 | 172 | 68 | 7 | 105586 |
| Chr01 | 92673260 | 92769506 | 172 | 559.3 | 172 | 80 | 2 | 68764 |
| Chr01 | 92769506 | 92786124 | 172 | 97.1 | 172 | 85 | 1 | 13845 |
| Chr01 | 93065593 | 93071681 | 172 | 35.4 | 172 | 87 | 0 | 5230 |
| Chr01 | 93071671 | 93080591 | 172 | 51.9 | 172 | 88 | 0 | 7781 |
| Chr01 | 93346339 | 93445337 | 172 | 575.6 | 172 | 87 | 0 | 85133 |
| Chr01 | 94049673 | 94063246 | 172 | 78.9 | 172 | 86 | 0 | 11451 |
| Chr01 | 94063164 | 94202191 | 172 | 804.2 | 172 | 57 | 11 | 20993 |
| Chr01 | 94152820 | 94202191 | 172 | 286.5 | 172 | 80 | 3 | 30509 |
| Chr02 | 81854 | 183852 | 172 | 593 | 172 | 86 | 0 | 85060 |
| Chr02 | 35479858 | 35592312 | 172 | 652.5 | 172 | 59 | 10 | 32820 |
| Chr02 | 40564965 | 40566759 | 172 | 10.4 | 172 | 77 | 2 | 1306 |
| Chr02 | 40566724 | 40572949 | 172 | 36.5 | 172 | 77 | 2 | 4446 |
| Chr02 | 40600512 | 40611134 | 172 | 62.8 | 168 | 56 | 11 | 2217 |
| Chr02 | 40611118 | 40618173 | 172 | 41.1 | 172 | 82 | 0 | 5656 |
| Chr02 | 40645705 | 40650379 | 172 | 27 | 172 | 67 | 6 | 1836 |
| Chr02 | 40650352 | 40654832 | 173 | 26 | 173 | 70 | 2 | 3014 |
| Chr02 | 40710739 | 40819145 | 173 | 625.7 | 173 | 50 | 12 | 26534 |
| Chr02 | 40819139 | 40848581 | 173 | 169.3 | 173 | 50 | 11 | 8216 |
| Chr02 | 40901444 | 40905258 | 173 | 22.1 | 173 | 71 | 2 | 2650 |
| Chr02 | 40952603 | 41335738 | 172 | 2227.1 | 172 | 63 | 10 | 77615 |
| Chr02 | 41335739 | 41341056 | 172 | 30.9 | 172 | 88 | 0 | 4609 |
| Chr02 | 55555113 | 55558530 | 173 | 19.5 | 173 | 68 | 10 | 1658 |
| Chr02 | 87268958 | 87287957 | 172 | 110.7 | 172 | 66 | 8 | 5466 |
| Chr03 | 165864 | 168751 | 172 | 16.9 | 172 | 83 | 0 | 2384 |
| Chr03 | 543119 | 637228 | 172 | 547.9 | 170 | 74 | 7 | 31321 |
| Chr03 | 546014 | 626470 | 172 | 468 | 172 | 77 | 4 | 40352 |
| Chr03 | 637253 | 648243 | 172 | 63.9 | 172 | 82 | 1 | 8847 |
| Chr03 | 648221 | 695351 | 172 | 274.1 | 172 | 84 | 0 | 38763 |
| Chr03 | 696156 | 709878 | 172 | 79.8 | 172 | 86 | 0 | 11690 |
| Chr03 | 20124632 | 20150410 | 172 | 149.8 | 172 | 79 | 2 | 18253 |
| Chr03 | 57536589 | 57538473 | 173 | 11.1 | 173 | 67 | 6 | 1120 |
| Chr04 | 547043 | 558551 | 172 | 67.1 | 172 | 87 | 0 | 9908 |
| Chr04 | 558549 | 566100 | 172 | 44.1 | 172 | 85 | 1 | 6375 |
| Chr04 | 822529 | 932527 | 172 | 639.9 | 172 | 84 | 1 | 84056 |
| Chr04 | 17355778 | 17382694 | 172 | 156.3 | 172 | 76 | 4 | 15316 |
| Chr04 | 17387102 | 17391044 | 172 | 22.9 | 172 | 85 | 0 | 3298 |
| Chr04 | 17393179 | 17450032 | 172 | 330.3 | 172 | 74 | 4 | 32772 |
| Chr04 | 17453217 | 17462390 | 172 | 53.2 | 172 | 84 | 1 | 7235 |
| Chr04 | 19680668 | 19748335 | 172 | 392.4 | 172 | 74 | 5 | 34764 |
| Chr04 | 19748326 | 19756877 | 172 | 50.1 | 172 | 76 | 4 | 5400 |
| Chr04 | 19950324 | 19956079 | 173 | 33.3 | 173 | 67 | 5 | 3334 |
| Chr04 | 19955483 | 19959957 | 173 | 25.8 | 173 | 69 | 2 | 2974 |
| Chr04 | 19967301 | 19969841 | 173 | 14.7 | 172 | 72 | 3 | 1764 |
| Chr04 | 27097053 | 27106253 | 172 | 53.4 | 172 | 63 | 9 | 3661 |
| Chr04 | 27125362 | 27135342 | 172 | 58 | 172 | 83 | 1 | 8003 |
| Chr04 | 37475501 | 37512477 | 172 | 215.1 | 172 | 79 | 2 | 25282 |
| Chr04 | 37512454 | 37514308 | 172 | 10.8 | 172 | 83 | 0 | 1505 |
| Chr04 | 37514285 | 37522831 | 172 | 49.9 | 172 | 80 | 1 | 6641 |
| Chr04 | 65434250 | 65628067 | 172 | 1124.9 | 172 | 71 | 6 | 70770 |
| Chr04 | 65634045 | 65639355 | 172 | 30.9 | 172 | 88 | 0 | 4600 |
| Chr04 | 65639342 | 65642132 | 172 | 16.2 | 172 | 87 | 0 | 2330 |
| Chr04 | 65646567 | 66137810 | 172 | 2854.9 | 172 | 70 | 6 | 188688 |
| Chr04 | 65820523 | 66320383 | 172 | 3121 | 155 | 43 | 16 | 25112 |
| Chr04 | 66137808 | 66607543 | 172 | 2726.8 | 172 | 65 | 9 | 89050 |
| Chr04 | 66608145 | 66664543 | 172 | 327.2 | 172 | 68 | 6 | 30224 |
| Chr04 | 66664644 | 66953959 | 172 | 1682.4 | 172 | 76 | 5 | 116376 |
| Chr04 | 66954061 | 67357986 | 172 | 2350 | 172 | 74 | 5 | 163418 |
| Chr04 | 67357957 | 67388178 | 172 | 175.7 | 172 | 87 | 0 | 26206 |
| Chr04 | 67388163 | 67407601 | 172 | 112.7 | 172 | 75 | 4 | 11765 |
| Chr04 | 67414156 | 67417871 | 172 | 21.6 | 172 | 83 | 0 | 2985 |
| Chr04 | 67417866 | 67420082 | 172 | 12.9 | 172 | 82 | 0 | 1772 |
| Chr04 | 67420077 | 67422292 | 172 | 12.9 | 172 | 82 | 0 | 1759 |
| Chr04 | 67422287 | 67424503 | 172 | 12.9 | 172 | 83 | 0 | 1768 |
| Chr04 | 67425302 | 67436759 | 172 | 66.5 | 172 | 79 | 1 | 8756 |
| Chr04 | 67438117 | 67508932 | 172 | 411.2 | 172 | 52 | 12 | 13273 |
| Chr04 | 70717816 | 70723704 | 173 | 34 | 173 | 71 | 1 | 4115 |
| Chr04 | 70729701 | 70731562 | 173 | 10.8 | 173 | 71 | 0 | 1311 |
| Chr04 | 70782515 | 70784759 | 173 | 13.1 | 173 | 70 | 2 | 1524 |
| Chr04 | 70792196 | 70795622 | 173 | 19.8 | 173 | 71 | 1 | 2391 |
| Chr04 | 70846915 | 70983580 | 173 | 787.4 | 173 | 52 | 11 | 39676 |
| Chr04 | 70986336 | 70988267 | 173 | 11.2 | 173 | 70 | 2 | 1351 |
| Chr04 | 71042686 | 71070040 | 173 | 157.9 | 173 | 69 | 1 | 18393 |
| Chr04 | 71098186 | 71100116 | 173 | 11.2 | 173 | 71 | 2 | 1292 |
| Chr04 | 71232110 | 71347041 | 172 | 669.8 | 172 | 82 | 2 | 86501 |
| Chr04 | 71378342 | 71468340 | 172 | 524.1 | 172 | 86 | 1 | 74594 |
| Chr04 | 71790437 | 71848707 | 172 | 339.1 | 172 | 86 | 0 | 49592 |
| Chr04 | 71848711 | 71911547 | 172 | 366.2 | 172 | 77 | 4 | 39617 |
| Chr05 | 293209 | 324952 | 172 | 184.6 | 172 | 64 | 8 | 9798 |
| Chr05 | 72153760 | 72174820 | 172 | 122.5 | 172 | 87 | 0 | 17978 |
| Chr05 | 72174808 | 72177699 | 172 | 16.8 | 172 | 88 | 0 | 2519 |
| Chr05 | 72177683 | 72195909 | 172 | 106 | 172 | 87 | 0 | 15584 |
| Chr06 | 10742110 | 10743889 | 172 | 10.5 | 171 | 73 | 4 | 1097 |
| Chr06 | 21966403 | 21993019 | 172 | 154.6 | 172 | 86 | 0 | 22641 |
| Chr06 | 33865398 | 34219592 | 172 | 2059.7 | 172 | 76 | 4 | 197713 |
| Chr06 | 34220851 | 34287534 | 172 | 385.8 | 172 | 60 | 10 | 14359 |
| Chr06 | 34329649 | 34331594 | 172 | 11.3 | 172 | 76 | 1 | 1399 |
| Chr06 | 34331556 | 34333828 | 172 | 13.2 | 172 | 77 | 1 | 1584 |
| Chr06 | 34336684 | 34340195 | 172 | 20.4 | 172 | 76 | 2 | 2461 |
| Chr06 | 34340381 | 34342560 | 172 | 12.6 | 172 | 77 | 1 | 1583 |
| Chr06 | 67190380 | 67223041 | 172 | 190.4 | 172 | 84 | 1 | 26527 |
| Chr06 | 67223038 | 67237272 | 172 | 82.8 | 172 | 85 | 0 | 12078 |
| Chr06 | 67237373 | 67252897 | 172 | 90.2 | 172 | 87 | 0 | 13305 |
| Chr06 | 67275982 | 67763117 | 172 | 2833.1 | 172 | 71 | 7 | 82974 |
| Chr06 | 67763121 | 67862349 | 172 | 577.1 | 172 | 68 | 8 | 34286 |
| Chr06 | 67847199 | 67925369 | 172 | 461.5 | 158 | 62 | 12 | 21341 |
| Chr06 | 67862332 | 67925371 | 172 | 367.1 | 172 | 86 | 0 | 53411 |
| Chr06 | 67925472 | 68013942 | 172 | 514.3 | 172 | 82 | 2 | 57927 |
| Chr06 | 68013947 | 68073202 | 172 | 344.7 | 172 | 87 | 0 | 50875 |
| Chr07 | 353329 | 434327 | 172 | 471.7 | 172 | 81 | 2 | 58570 |
| Chr07 | 2108559 | 2608558 | 172 | 2905.5 | 172 | 76 | 4 | 256539 |
| Chr07 | 2607875 | 2803398 | 172 | 1134.1 | 172 | 79 | 3 | 133129 |
| Chr07 | 2803396 | 3271442 | 172 | 2721.2 | 172 | 65 | 9 | 75866 |
| Chr07 | 3131631 | 3376541 | 172 | 1433.6 | 162 | 78 | 6 | 141624 |
| Chr07 | 3376642 | 3859338 | 172 | 2803.2 | 172 | 69 | 7 | 194694 |
| Chr07 | 3861088 | 3972902 | 173 | 645.6 | 172 | 63 | 4 | 61799 |
| Chr07 | 4318006 | 4320094 | 173 | 12.1 | 174 | 68 | 1 | 1421 |
| Chr07 | 4339629 | 4344092 | 173 | 25.7 | 173 | 48 | 11 | 1257 |
| Chr07 | 4345928 | 4348368 | 173 | 14.1 | 173 | 69 | 1 | 1622 |
| Chr07 | 4361865 | 4366122 | 173 | 24.6 | 172 | 67 | 1 | 2828 |
| Chr07 | 4366119 | 4368598 | 173 | 14.3 | 172 | 68 | 1 | 1651 |
| Chr07 | 4510550 | 4523960 | 173 | 77.1 | 172 | 57 | 7 | 5750 |
| Chr07 | 4652971 | 4656159 | 173 | 18.4 | 173 | 70 | 2 | 2090 |
| Chr07 | 4656114 | 4664051 | 173 | 46 | 173 | 47 | 14 | 1600 |
| Chr07 | 4672024 | 4673785 | 173 | 10.2 | 172 | 69 | 3 | 1138 |
| Chr07 | 4689591 | 4775084 | 173 | 494.1 | 172 | 49 | 12 | 22892 |
| Chr07 | 4777230 | 4780515 | 173 | 19 | 172 | 69 | 3 | 2170 |
| Chr07 | 4808842 | 4930025 | 172 | 704.7 | 172 | 76 | 5 | 56874 |
| Chr07 | 66441765 | 66490245 | 172 | 282.2 | 172 | 86 | 1 | 40630 |
| Chr07 | 66490247 | 66544752 | 172 | 317 | 172 | 73 | 5 | 24880 |
| Chr08 | 26956393 | 26958609 | 173 | 12.8 | 172 | 71 | 3 | 1540 |
| Chr08 | 62744387 | 62751214 | 172 | 39.4 | 172 | 63 | 8 | 2597 |
| Chr08 | 62751210 | 62762883 | 172 | 67.9 | 172 | 80 | 0 | 9002 |
| Chr08 | 62769828 | 62792407 | 172 | 131 | 172 | 63 | 7 | 7871 |
| Chr08 | 62795763 | 62860508 | 172 | 375.3 | 172 | 57 | 10 | 13690 |
| Chr08 | 62860506 | 63010843 | 172 | 873.5 | 172 | 53 | 12 | 21223 |
| Chr08 | 63011270 | 63015393 | 172 | 24 | 172 | 82 | 0 | 3266 |
| Chr08 | 63015393 | 63024348 | 172 | 52 | 172 | 72 | 4 | 4977 |
| Chr08 | 63024508 | 63161445 | 172 | 794.8 | 172 | 60 | 10 | 29008 |
| Chr08 | 63181323 | 63189377 | 172 | 46.8 | 172 | 66 | 6 | 3551 |
| Chr08 | 63195481 | 63431897 | 172 | 1371.9 | 172 | 57 | 10 | 55392 |
| Chr08 | 63431894 | 63433906 | 172 | 11.7 | 172 | 81 | 1 | 1569 |
| Chr08 | 63446908 | 63458141 | 172 | 65.2 | 172 | 54 | 11 | 1811 |
| Chr08 | 63458420 | 63735685 | 172 | 1610.2 | 172 | 61 | 9 | 77039 |
| Chr08 | 63735671 | 63745350 | 172 | 56.3 | 172 | 81 | 0 | 7637 |
| Chr08 | 63745325 | 63750207 | 172 | 28.4 | 172 | 81 | 0 | 3820 |
| Chr08 | 64257183 | 64261826 | 172 | 27 | 172 | 82 | 0 | 3704 |
| Chr08 | 64261819 | 64274656 | 172 | 74.7 | 172 | 81 | 0 | 10091 |
| Chr09 | 2101 | 4100 | 172 | 11.6 | 172 | 85 | 0 | 1694 |
| Chr09 | 156468 | 271901 | 172 | 670.9 | 172 | 76 | 4 | 64412 |
| Chr09 | 1200584 | 1204378 | 173 | 21.9 | 172 | 68 | 2 | 2531 |
| Chr09 | 1486123 | 1493064 | 172 | 40.1 | 172 | 56 | 10 | 2347 |
| Chr09 | 1493054 | 1496922 | 172 | 22.5 | 172 | 81 | 0 | 3030 |
| Chr09 | 1987760 | 1990588 | 172 | 16.5 | 172 | 87 | 0 | 2438 |
| Chr09 | 1990940 | 2005956 | 172 | 87.4 | 172 | 80 | 2 | 11228 |
| Chr09 | 2005944 | 2035809 | 172 | 173.5 | 172 | 73 | 4 | 18433 |
| Chr09 | 1996289 | 2035810 | 172 | 231.3 | 167 | 68 | 7 | 14224 |
| Chr09 | 49789103 | 49954777 | 173 | 953.2 | 173 | 50 | 12 | 29357 |
| Chr09 | 49955825 | 50062469 | 173 | 615.9 | 173 | 56 | 8 | 43529 |
| Chr09 | 50203976 | 50236061 | 173 | 186.2 | 172 | 64 | 4 | 17720 |
| Chr09 | 50313048 | 50324654 | 173 | 67.1 | 172 | 69 | 2 | 7741 |
| Chr09 | 50324652 | 50329390 | 173 | 27.4 | 173 | 69 | 1 | 3177 |
| Chr09 | 50410956 | 50414578 | 173 | 21 | 172 | 69 | 3 | 2421 |
| Chr09 | 50418113 | 50869106 | 172 | 2612.9 | 172 | 59 | 11 | 56055 |
| Chr09 | 50870013 | 50984241 | 172 | 662.9 | 172 | 78 | 3 | 71322 |
| Chr09 | 50903681 | 51090095 | 172 | 1060.4 | 180 | 57 | 11 | 43321 |
| Chr09 | 51703306 | 51714303 | 172 | 64 | 172 | 52 | 11 | 2568 |
| Chr09 | 52093457 | 52096456 | 172 | 17.5 | 172 | 88 | 0 | 2590 |
| Chr09 | 52440400 | 52491399 | 172 | 296.9 | 172 | 84 | 1 | 37779 |
| Chr09 | 52491500 | 52522736 | 172 | 181.7 | 172 | 87 | 0 | 26844 |
| Chr10 | 49636722 | 49770136 | 172 | 775.3 | 172 | 81 | 3 | 88903 |
| Chr10 | 49770117 | 49796663 | 172 | 154.4 | 172 | 87 | 0 | 22784 |
| Chr10 | 49842276 | 49887964 | 172 | 265.9 | 172 | 88 | 0 | 39604 |
| Chr10 | 50370726 | 50404638 | 173 | 196.3 | 173 | 68 | 2 | 22470 |
| Chr11 | 303881 | 446497 | 172 | 827.8 | 172 | 69 | 7 | 53195 |
| Chr11 | 446491 | 448529 | 172 | 11.9 | 172 | 88 | 0 | 1777 |
| Chr11 | 449054 | 451746 | 172 | 15.6 | 172 | 88 | 0 | 2303 |
| Chr11 | 451733 | 900122 | 172 | 2603.3 | 172 | 64 | 9 | 61920 |
| Chr11 | 662091 | 1138073 | 172 | 2768.9 | 171 | 64 | 9 | 67834 |
| Chr11 | 900121 | 1367999 | 172 | 2720 | 172 | 67 | 8 | 130633 |
| Chr11 | 987511 | 1487510 | 172 | 2955.7 | 161 | 68 | 10 | 190435 |
| Chr11 | 1373024 | 1821044 | 172 | 2606.3 | 171 | 67 | 8 | 88939 |
| Chr11 | 1821147 | 2079924 | 172 | 1504.1 | 172 | 77 | 4 | 124182 |
| Chr11 | 2081961 | 2088476 | 172 | 37.9 | 172 | 89 | 0 | 5735 |
| Chr11 | 2094971 | 2153949 | 172 | 342.2 | 172 | 71 | 6 | 27107 |
| Chr11 | 2154071 | 2160162 | 172 | 35.6 | 172 | 85 | 0 | 5143 |
| Chr11 | 2160194 | 2206542 | 172 | 269.1 | 172 | 87 | 1 | 38944 |
| Chr11 | 2206529 | 2282969 | 172 | 443.9 | 172 | 76 | 5 | 40459 |
| Chr11 | 41599977 | 41799760 | 172 | 1161.1 | 172 | 75 | 4 | 105797 |
| Chr11 | 41799749 | 41824308 | 172 | 143.2 | 172 | 82 | 1 | 18632 |
| Chr11 | 42063721 | 42068720 | 172 | 29.1 | 172 | 88 | 0 | 4312 |
| Chr13 | 1286976 | 1335666 | 172 | 284 | 172 | 76 | 4 | 30429 |
| Chr13 | 6668698 | 6673006 | 172 | 25.1 | 172 | 73 | 1 | 2864 |
| Chr13 | 6693790 | 6697383 | 172 | 21 | 172 | 74 | 2 | 2470 |
| Chr13 | 25674063 | 25677979 | 172 | 22.8 | 172 | 88 | 0 | 3444 |
| Chr13 | 25685011 | 25699281 | 172 | 83.2 | 172 | 86 | 0 | 12204 |
| Chr13 | 25699265 | 25715859 | 172 | 96.3 | 172 | 76 | 3 | 11372 |
| Chr13 | 25772039 | 25943549 | 173 | 989.8 | 173 | 58 | 7 | 75168 |
| Chr13 | 28861123 | 29101142 | 172 | 1392.8 | 172 | 67 | 7 | 110793 |
| Chr13 | 29101143 | 29123001 | 172 | 126.8 | 172 | 76 | 5 | 11536 |
| Chr13 | 33489728 | 33648571 | 172 | 924.8 | 171 | 77 | 4 | 88528 |
| Chr13 | 33648563 | 33725766 | 172 | 449.7 | 172 | 85 | 0 | 64296 |
| Chr16 | 2841040 | 2861538 | 172 | 118.9 | 172 | 59 | 9 | 7616 |
| Chr16 | 7229441 | 7231440 | 172 | 11.6 | 172 | 85 | 0 | 1704 |
| Chr16 | 22711312 | 22734321 | 172 | 133.6 | 172 | 68 | 7 | 7421 |
| Chr16 | 22736298 | 22743794 | 172 | 43.4 | 172 | 83 | 1 | 6112 |
| tig00000001326 | 1 | 21479 | 172 | 124.7 | 172 | 83 | 1 | 17065 |
| tig0000000641 | 1 | 35164 | 172 | 204.3 | 171 | 70 | 7 | 14308 |

# Supplementary Table 11. The putative telomeres on the *S. cusia* chromosomes.

| **Contig_Name** | **Contig_Size** | **Start_of_telo** | **End_of_telo** | **Size_of_telo** | **Teleomeric_repeat_sequence** | **posi_of_telo** | **No_of_tel** |
| --- | --- | --- | --- | --- | --- | --- | --- |
| Chr01 | 94747430 | 4678138 | 4681346 | 3208 | ACCCTAA | start | 458.7 |
| Chr01 | 94747430 | 90415765 | 90418873 | 3108 | AAACCCT | end | 443.9 |
| Chr02 | 87398577 | 40943587 | 40947856 | 4269 | TAGGGTT | start | 613.6 |
| Chr02 | 87398577 | 61136924 | 61140162 | 3238 | TTAGGGT | end | 462.7 |
| Chr02 | 87398577 | 80574242 | 80577766 | 3524 | GGGTTTA | end | 505.1 |
| Chr03 | 77968717 | 849099 | 852643 | 3544 | ACCCTAA | start | 506 |
| Chr03 | 77968717 | 53416229 | 53418644 | 2415 | CCTAAAC | end | 344.9 |
| Chr04 | 72211168 | 23313877 | 23318158 | 4281 | TAGGGTT | start | 611.9 |
| Chr04 | 72211168 | 23318259 | 23321441 | 3182 | AAACCCT | start | 456.3 |
| Chr04 | 72211168 | 65257843 | 65260195 | 2352 | TAGGGTT | end | 345.1 |
| Chr04 | 72211168 | 71030476 | 71033484 | 3008 | TAGGGTT | end | 437 |
| Chr05 | 72205009 | 56157340 | 56160567 | 3227 | AACCCTA | end | 461 |
| Chr06 | 68439447 | 64538036 | 64542069 | 4033 | ACCCTAA | end | 576.3 |
| Chr07 | 66544764 | 51876886 | 51880094 | 3208 | GGGTTTA | end | 462.3 |
| Chr07 | 66544764 | 52504038 | 52506968 | 2930 | AACCCTA | end | 419.9 |
| Chr08 | 64376749 | 26971802 | 26974898 | 3096 | GGTTTAG | start | 444.1 |
| Chr08 | 64376749 | 27050799 | 27054612 | 3813 | ACCCTAA | start | 544.4 |
| Chr08 | 64376749 | 27050816 | 27055125 | 4309 | CTGAACCCTAAACCCAAGTATTCATGCTCCCAGAAACCCTATT | start | 102.6 |
| Chr09 | 52549036 | 49755296 | 49758598 | 3302 | GGTTTAG | end | 472 |
| Chr10 | 50440415 | 48009149 | 48012113 | 2964 | GTTTAGG | end | 424 |
| Chr11 | 42103120 | 2389355 | 2392824 | 3469 | TAAACCC | start | 496.7 |
| Chr11 | 42103120 | 41123193 | 41126791 | 3598 | GGGTTTA | end | 515.3 |
| Chr12 | 38095908 | 29223828 | 29226225 | 2397 | TAGGGTT | end | 342.6 |
| Chr13 | 35037375 | 25630705 | 25632543 | 1838 | AGGGTTT | end | 269.6 |
| Chr13 | 35037375 | 25762160 | 25764804 | 2644 | CCTAAAC | end | 376.9 |
| Chr13 | 35037375 | 30542104 | 30544257 | 2153 | GGGTTTA | end | 307.4 |
| Chr13 | 35037375 | 30773531 | 30775664 | 2133 | CCTAAAC | end | 305.3 |
| Chr16 | 22812488 | 21921940 | 21925820 | 3880 | ACCCTAA | end | 557.7 |
| Chr16 | 22812488 | 22423516 | 22426405 | 2889 | ACCCTAA | end | 420.9 |

# Supplementary Table 12. Statistical analysis of gene family cluster.

| **Species** | **Proteins** | **Clusters** | **Singletons** |
| --- | --- | --- | --- |
| *S. cusia* | 32974 | 16955 | 6503 |
| *A. paniculata* | 25428 | 14074 | 7794 |
| *M. guttatus* | 33573 | 17270 | 5500 |
| *O. europaea* var*. sylvestris* | 50684 | 16321 | 13500 |
| *S. indicum* | 35410 | 17113 | 2559 |
| *S. splendens* | 53354 | 17950 | 9412 |
| Total | 231423 | 99683 | 45268 |

# Supplementary Table 13. GO enrichment analysis of the gene family significantly (FDR < 0.05) specific to the *S. cusia* genome.

| GO ID | Count | Name | Type | *P*-value |
| --- | --- | --- | --- | --- |
| GO:0015074 | 26 | DNA integration | biological_process | 1.86E-27 |
| GO:0009626 | 17 | plant-type hypersensitive response | biological_process | 2.37E-12 |
| GO:0032197 | 8 | transposition, RNA-mediated | biological_process | 8.34E-10 |
| GO:0006952 | 24 | defense response | biological_process | 2.02E-09 |
| GO:0006310 | 9 | DNA recombination | biological_process | 8.00E-09 |
| GO:0032196 | 5 | transposition | biological_process | 5.10E-07 |
| GO:0050832 | 8 | defense response to fungus | biological_process | 8.65E-07 |
| GO:0080142 | 5 | regulation of salicylic acid biosynthetic process | biological_process | 1.07E-05 |
| GO:0009791 | 4 | post-embryonic development | biological_process | 0.000714 |
| GO:0008137 | 2 | NADH dehydrogenase (ubiquinone) activity | molecular_function | 0.001344 |
| GO:0004129 | 2 | cytochrome-c oxidase activity | molecular_function | 0.001344 |
| GO:0047172 | 2 | shikimate O-hydroxycinnamoyltransferase activity | molecular_function | 0.002631 |

# Supplementary Table 14. GO (Biological Process) enrichment analysis of the gene family with significant expansion to the *S. cusia* genome

| GO ID | Description | Number of genes | *P*-value |
| --- | --- | --- | --- |
| GO:0080088 | spermidine hydroxycinnamate conjugate biosynthetic process | 6 | 2.34E-12 |
| GO:0019742 | pentacyclic triterpenoid metabolic process | 4 | 2.50E-08 |
| GO:0019745 | pentacyclic triterpenoid biosynthetic process | 4 | 2.50E-08 |
| GO:0009873 | ethylene-activated signaling pathway | 10 | 2.60E-08 |
| GO:0006355 | regulation of transcription, DNA-templated | 40 | 2.61E-08 |
| GO:0010468 | regulation of gene expression | 45 | 2.69E-08 |
| GO:1903506 | regulation of nucleic acid-templated transcription | 40 | 4.27E-08 |
| GO:2001141 | regulation of RNA biosynthetic process | 40 | 4.27E-08 |
| GO:0006351 | transcription, DNA-templated | 40 | 8.50E-08 |
| GO:0071369 | cellular response to ethylene stimulus | 10 | 9.80E-08 |
| GO:0097659 | nucleic acid-templated transcription | 40 | 1.30E-07 |
| GO:0051252 | regulation of RNA metabolic process | 40 | 1.41E-07 |
| GO:0032774 | RNA biosynthetic process | 40 | 1.65E-07 |
| GO:0016103 | diterpenoid catabolic process | 5 | 1.68E-07 |
| GO:0045487 | gibberellin catabolic process | 5 | 1.68E-07 |
| GO:0016104 | triterpenoid biosynthetic process | 4 | 1.73E-07 |
| GO:0000160 | phosphorelay signal transduction system | 10 | 2.04E-07 |
| GO:0034654 | nucleobase-containing compound biosynthetic process | 43 | 3.01E-07 |
| GO:0044271 | cellular nitrogen compound biosynthetic process | 50 | 3.04E-07 |
| GO:1901362 | organic cyclic compound biosynthetic process | 47 | 3.87E-07 |

**Supplementary Table 15. KEGG pathway of expansion gene families in *S. cusia*.**

| KEGG_B_class | Pathway | Number of genes | *P*-value | Pathway ID |
| --- | --- | --- | --- | --- |
| Signal transduction | MAPK signaling pathway - plant | 23 | 1.33E-18 | ko04016 |
| Environmental adaptation | Plant-pathogen interaction | 26 | 3.42E-14 | ko04626 |
| Metabolism of terpenoids and polyketides | Diterpenoid biosynthesis | 5 | 1.41E-05 | ko00904 |
| Metabolism of terpenoids and polyketides | Carotenoid biosynthesis | 4 | 0.000513 | ko00906 |
| Metabolism of terpenoids and polyketides | Sesquiterpenoid and triterpenoid biosynthesis | 2 | 0.014358 | ko00909 |
| Lipid metabolism | Steroid biosynthesis | 2 | 0.029228 | ko00100 |

# Supplementary Table 17. bHLH gene family information and physical and chemical properties.

| **bHLH ID** | **Gene accession** | **Chromosome name** | **Strand** | **Protein length (aa)** | **Isoelectric point** | **Molecular mass/kDa** |
| --- | --- | --- | --- | --- | --- | --- |
| MLbHLH1 | ML11582 | Chr04 | + | 369 | 5.2 | 40126.97 |
| MLbHLH2 | ML19205 | Chr07 | - | 340 | 6.41 | 37606.56 |
| MLbHLH3 | ML00047 | Chr04 | + | 417 | 5.19 | 45866.69 |
| MLbHLH4 | ML08896 | Chr02 | - | 306 | 7.93 | 33736.61 |
| MLbHLH5 | ML09337 | Chr10 | - | 307 | 6.87 | 33748.38 |
| MLbHLH6 | ML25693 | Chr06 | + | 322 | 7.05 | 35562.37 |
| MLbHLH7 | ML27451 | Chr09 | + | 343 | 6.77 | 37914.91 |
| MLbHLH8 | ML03443 | Chr03 | + | 355 | 5.11 | 40015.4 |
| MLbHLH9 | ML07536 | Chr16 | - | 375 | 5.61 | 41568.05 |
| MLbHLH10 | ML12235 | Chr06 | + | 349 | 4.75 | 38932.39 |
| MLbHLH11 | ML08650 | Chr02 | - | 273 | 10.41 | 31719.52 |
| MLbHLH12 | ML19973 | Chr07 | - | 237 | 10.25 | 26751.38 |
| MLbHLH13 | ML32670 | Chr08 | - | 535 | 5.35 | 59013.91 |
| MLbHLH14 | ML06447 | Chr04 | - | 429 | 8.26 | 48252.2 |
| MLbHLH15 | ML03567 | Chr03 | - | 224 | 10.09 | 24687.48 |
| MLbHLH16 | ML17026 | Chr14 | + | 239 | 5.71 | 26307.73 |
| MLbHLH17 | ML25335 | Chr05 | - | 252 | 6.59 | 27944.37 |
| MLbHLH18 | ML08003 | Chr15 | + | 388 | 6.8 | 42780.86 |
| MLbHLH19 | ML30647 | Chr05 | - | 364 | 5.76 | 40200.94 |
| MLbHLH20 | ML12253 | Chr06 | - | 344 | 8.12 | 36817.74 |
| MLbHLH21 | ML03517 | Chr03 | + | 366 | 4.63 | 40205.33 |
| MLbHLH22 | ML07477 | Chr16 | - | 346 | 5.05 | 38336.73 |
| MLbHLH23 | ML17320 | Chr14 | - | 422 | 4.94 | 45816.97 |
| MLbHLH24 | ML04220 | Chr03 | + | 338 | 4.72 | 37411.73 |
| MLbHLH25 | ML03186 | Chr03 | + | 265 | 4.64 | 28517.46 |
| MLbHLH26 | ML01935 | Chr01 | - | 290 | 6.65 | 31477.11 |
| MLbHLH27 | ML00484 | Chr01 | - | 273 | 6.51 | 30321.51 |
| MLbHLH28 | ML03495 | Chr03 | + | 420 | 6.2 | 46124.99 |
| MLbHLH29 | ML07504 | Chr16 | - | 370 | 5.5 | 39590.7 |
| MLbHLH30 | ML24431 | Chr02 | + | 388 | 6.86 | 42343.48 |
| MLbHLH31 | ML01470 | Chr08 | - | 355 | 5.12 | 38974.16 |
| MLbHLH32 | ML01034 | Chr08 | - | 316 | 6.99 | 33752.7 |
| MLbHLH33 | ML04430 | Chr03 | + | 296 | 6.73 | 32191.16 |
| MLbHLH34 | ML21594 | Chr16 | + | 453 | 8.9 | 49581.27 |
| MLbHLH35 | ML30699 | Chr05 | - | 314 | 6.77 | 33359.1 |
| MLbHLH36 | ML05975 | Chr04 | + | 290 | 6.5 | 32677.23 |
| MLbHLH37 | ML22145 | Chr02 | + | 424 | 6.89 | 44076.47 |
| MLbHLH38 | ML18662 | Chr11 | + | 432 | 6.33 | 48310.71 |
| MLbHLH39 | ML06572 | Chr04 | - | 384 | 7.61 | 41426.08 |
| MLbHLH40 | ML16300 | Chr02 | + | 341 | 6.09 | 37203.1 |
| MLbHLH41 | ML26077 | Chr01 | + | 310 | 7.15 | 33687.88 |
| MLbHLH42 | ML06204 | Chr04 | + | 514 | 7.57 | 55601.6 |
| MLbHLH43 | ML19727 | Chr07 | - | 439 | 7.64 | 48154.82 |
| MLbHLH44 | ML32106 | Chr02 | - | 669 | 5.39 | 73199.88 |
| MLbHLH45 | ML03833 | Chr03 | + | 391 | 7 | 43997.36 |
| MLbHLH46 | ML12359 | Chr06 | - | 293 | 7.11 | 32507.67 |
| MLbHLH47 | ML00529 | Chr08 | - | 304 | 4.5 | 34470.56 |
| MLbHLH48 | ML19823 | Chr07 | - | 314 | 4.76 | 35423.54 |
| MLbHLH49 | ML10727 | Chr04 | - | 222 | 7.43 | 24715.89 |
| MLbHLH50 | ML16721 | Chr02 | - | 238 | 8.7 | 26030.99 |
| MLbHLH51 | ML06382 | Chr04 | - | 281 | 7.63 | 30789 |
| MLbHLH52 | ML14461 | Chr06 | - | 279 | 7.67 | 30923.9 |
| MLbHLH53 | ML18016 | Chr14 | + | 272 | 9.25 | 30153.2 |
| MLbHLH54 | ML18993 | Chr07 | + | 517 | 6.13 | 56045.84 |
| MLbHLH55 | ML08573 | Chr15 | + | 189 | 8.72 | 21450.16 |
| MLbHLH56 | ML20093 | Chr07 | + | 204 | 8.24 | 22671.54 |
| MLbHLH57 | ML05967 | Chr04 | - | 498 | 7.37 | 56261.35 |
| MLbHLH58 | ML05968 | Chr04 | - | 475 | 6.33 | 53525.17 |
| MLbHLH59 | ML08522 | Chr15 | + | 234 | 5.19 | 25938.46 |
| MLbHLH60 | ML30369 | Chr05 | + | 589 | 6.18 | 64860.99 |
| MLbHLH61 | ML01111 | Chr08 | + | 464 | 8.23 | 52432.24 |
| MLbHLH62 | ML10488 | Chr06 | - | 563 | 7.96 | 63288.6 |
| MLbHLH63 | ML25557 | Chr06 | + | 238 | 6.72 | 26725.86 |
| MLbHLH64 | ML32810 | Chr08 | - | 527 | 6.46 | 57871.03 |
| MLbHLH65 | ML15096 | Chr09 | - | 306 | 6.84 | 34336.82 |
| MLbHLH66 | ML07343 | Chr14 | + | 199 | 10.67 | 22687.32 |
| MLbHLH67 | ML28096 | Chr02 | - | 226 | 8.23 | 25536.12 |
| MLbHLH68 | ML01235 | Chr08 | + | 241 | 5.06 | 26991.75 |
| MLbHLH69 | ML17119 | Chr14 | + | 275 | 5.09 | 31207.3 |
| MLbHLH70 | ML17121 | Chr14 | + | 275 | 4.76 | 31154.15 |
| MLbHLH71 | ML00905 | Chr08 | - | 162 | 4.51 | 18089.22 |
| MLbHLH72 | ML26436 | Chr09 | + | 257 | 6.42 | 28296.11 |
| MLbHLH73 | ML29147 | Chr01 | - | 253 | 6.9 | 27863.02 |
| MLbHLH74 | ML00349 | Chr15 | - | 372 | 7.32 | 41316.46 |
| MLbHLH75 | ML00448 | Chr01 | + | 363 | 5.92 | 39549.4 |
| MLbHLH76 | ML15463 | Chr11 | + | 255 | 8.96 | 28003.94 |
| MLbHLH77 | ML24782 | Chr13 | + | 233 | 10.17 | 25667.84 |
| MLbHLH78 | ML25712 | Chr06 | + | 259 | 8.36 | 28293.63 |
| MLbHLH79 | ML27057 | Chr09 | + | 200 | 10.71 | 21865.22 |
| MLbHLH80 | ML28571 | Chr11 | + | 266 | 5.85 | 29328.34 |
| MLbHLH81 | ML11764 | Chr15 | - | 489 | 6.07 | 53846.47 |
| MLbHLH82 | ML17582 | Chr14 | + | 499 | 5.59 | 55611.49 |
| MLbHLH83 | ML00088 | Chr04 | + | 325 | 5.93 | 36912.91 |
| MLbHLH84 | ML04185 | Chr03 | + | 345 | 8.07 | 38631.16 |
| MLbHLH85 | ML06412 | Chr04 | - | 280 | 5.72 | 31620.37 |
| MLbHLH86 | ML06414 | Chr04 | - | 280 | 5.96 | 31592.35 |
| MLbHLH87 | ML07252 | Chr14 | + | 353 | 6.33 | 39299.09 |
| MLbHLH88 | ML17340 | Chr14 | + | 312 | 8.2 | 34772.22 |
| MLbHLH89 | ML07339 | Chr14 | + | 341 | 5.88 | 37958.36 |
| MLbHLH90 | ML17034 | Chr14 | + | 295 | 6.37 | 31231.84 |
| MLbHLH91 | ML15623 | Chr11 | - | 195 | 5.12 | 22286.49 |
| MLbHLH92 | ML24822 | Chr13 | + | 601 | 5.3 | 67480.67 |
| MLbHLH93 | ML30784 | Chr05 | + | 324 | 5.31 | 36200.04 |
| MLbHLH94 | ML05430 | Chr04 | - | 191 | 8.64 | 21163.16 |
| MLbHLH95 | ML13087 | Chr06 | + | 197 | 7.13 | 21758.04 |
| MLbHLH96 | ML16738 | Chr02 | + | 179 | 9.08 | 19552.16 |
| MLbHLH97 | ML28630 | Chr11 | + | 233 | 9.53 | 25646.97 |
| MLbHLH98 | ML14089 | Chr06 | - | 103 | 7.92 | 11362.99 |
| MLbHLH99 | ML19272 | Chr07 | - | 95 | 8.66 | 10542.13 |
| MLbHLH100 | ML19273 | Chr07 | + | 93 | 10.32 | 10608.98 |
| MLbHLH101 | ML16571 | Chr02 | + | 341 | 7.04 | 37999.46 |
| MLbHLH102 | ML09083 | Chr02 | + | 477 | 8.44 | 51422.06 |
| MLbHLH103 | ML21442 | Chr16 | + | 507 | 7.73 | 55696.68 |
| MLbHLH104 | ML27711 | Chr02 | + | 457 | 7 | 48643.48 |
| MLbHLH105 | ML32723 | Chr08 | - | 394 | 8.24 | 42835.78 |
| MLbHLH106 | ML30301 | Chr05 | - | 651 | 6.4 | 71938.51 |
| MLbHLH107 | ML09731 | Chr10 | - | 191 | 5.87 | 21242.32 |
| MLbHLH108 | ML31395 | Chr02 | + | 238 | 6.85 | 26640.96 |
| MLbHLH109 | ML17531 | Chr14 | - | 478 | 7.17 | 52588.72 |
| MLbHLH110 | ML32207 | Chr02 | + | 371 | 7.33 | 41551.27 |
| MLbHLH111 | ML06340 | Chr04 | - | 406 | 7.54 | 44291.75 |
| MLbHLH112 | ML15185 | Chr09 | - | 332 | 9.01 | 36378.15 |
| MLbHLH113 | ML23940 | Chr02 | - | 423 | 7.95 | 45784.5 |
| MLbHLH114 | ML32654 | Chr08 | + | 383 | 8.02 | 41886.6 |
| MLbHLH115 | ML17505 | Chr14 | + | 602 | 7.1 | 66206 |
| MLbHLH116 | ML07801 | Chr13 | - | 301 | 8.64 | 32803.63 |
| MLbHLH117 | ML28095 | Chr02 | - | 264 | 4.88 | 29309.81 |
| MLbHLH118 | ML28097 | Chr02 | - | 254 | 8.24 | 28593.83 |
| MLbHLH119 | ML28098 | Chr02 | - | 249 | 7.53 | 27802.69 |
| MLbHLH120 | ML28747 | Chr11 | + | 464 | 6.06 | 50044.03 |
| MLbHLH121 | ML29458 | Chr05 | - | 379 | 7.69 | 40712.59 |
| MLbHLH122 | ML14413 | Chr06 | + | 339 | 8.7 | 37537.1 |
| MLbHLH123 | ML06586 | Chr04 | + | 325 | 6.52 | 36061.92 |
| MLbHLH124 | ML20188 | Chr07 | + | 294 | 6.53 | 32642.54 |
| MLbHLH125 | ML11463 | Chr04 | - | 250 | 6.65 | 28585.23 |
| MLbHLH126 | ML11464 | Chr04 | - | 235 | 8.52 | 26990.83 |
| MLbHLH127 | ML02628 | Chr01 | + | 246 | 8.82 | 27485.54 |
| MLbHLH128 | ML29912 | Chr05 | - | 262 | 6.03 | 28761.7 |
| MLbHLH129 | ML08261 | Chr15 | - | 482 | 5.03 | 52511.99 |
| MLbHLH130 | ML10608 | Chr15 | - | 584 | 5.64 | 62672.14 |
| MLbHLH131 | ML21691 | Chr16 | - | 449 | 4.75 | 49599.44 |
| MLbHLH132 | ML08351 | Chr15 | + | 337 | 7.91 | 36691.73 |
| MLbHLH133 | ML15620 | Chr11 | - | 384 | 6.26 | 41369.94 |
| MLbHLH134 | ML16788 | Chr02 | + | 402 | 7.56 | 44158.22 |
| MLbHLH135 | ML31316 | Chr02 | - | 377 | 4.7 | 41298.04 |
| MLbHLH136 | ML16898 | Chr14 | - | 461 | 7.58 | 50083 |
| MLbHLH137 | ML12661 | Chr06 | - | 238 | 8.61 | 26083.63 |
| MLbHLH138 | ML25010 | Chr11 | - | 240 | 8.61 | 26360.88 |
| MLbHLH139 | ML29437 | Chr12 | - | 236 | 8.61 | 26146.62 |
| MLbHLH140 | ML31426 | Chr02 | + | 206 | 10.78 | 22878.96 |
| MLbHLH141 | ML05617 | Chr04 | + | 321 | 5.78 | 36163.86 |
| MLbHLH142 | ML18996 | Chr07 | + | 335 | 6.07 | 37065.94 |
| MLbHLH143 | ML26517 | Chr09 | + | 333 | 5.36 | 36733.74 |
| MLbHLH144 | ML09904 | Chr10 | + | 644 | 6.19 | 71305.02 |
| MLbHLH145 | ML31920 | Chr02 | + | 639 | 6.12 | 70846.93 |
| MLbHLH146 | ML04120 | Chr03 | - | 108 | 10.49 | 12040.57 |
| MLbHLH147 | ML04270 | Chr03 | - | 285 | 7.74 | 32024.98 |
| MLbHLH148 | ML05065 | Chr03 | + | 548 | 9.38 | 60042.79 |
| MLbHLH149 | ML05658 | Chr04 | + | 103 | 10.12 | 11343.75 |
| MLbHLH150 | ML07870 | Chr13 | + | 277 | 5.52 | 31151.39 |
| MLbHLH151 | ML10633 | Chr15 | + | 280 | 7.21 | 31394.28 |
| MLbHLH152 | ML11301 | Chr04 | + | 305 | 5.58 | 33300.73 |
| MLbHLH153 | ML11937 | Chr06 | + | 101 | 10.21 | 11039.34 |
| MLbHLH154 | ML11941 | Chr06 | - | 308 | 7.69 | 34205.21 |
| MLbHLH155 | ML14908 | Chr09 | - | 93 | 8.44 | 10579.87 |
| MLbHLH156 | ML15855 | Chr05 | + | 234 | 5.35 | 26551.57 |
| MLbHLH157 | ML16673 | Chr02 | + | 717 | 7.11 | 76065.99 |
| MLbHLH158 | ML17286 | Chr14 | + | 340 | 6.78 | 37586.32 |
| MLbHLH159 | ML17591 | Chr14 | + | 296 | 6.52 | 31458.29 |
| MLbHLH160 | ML17940 | Chr14 | - | 308 | 6 | 32517.96 |
| MLbHLH161 | ML17972 | Chr14 | - | 518 | 6.26 | 56922.29 |
| MLbHLH162 | ML19092 | Chr07 | + | 93 | 10.21 | 10548.9 |
| MLbHLH163 | ML19939 | Chr07 | + | 241 | 5.83 | 27262.95 |
| MLbHLH164 | ML21464 | Chr16 | - | 547 | 7.58 | 59569.02 |
| MLbHLH165 | ML21704 | Chr16 | + | 353 | 9.92 | 39162.41 |
| MLbHLH166 | ML21880 | Chr02 | - | 339 | 6.62 | 37311.27 |
| MLbHLH167 | ML24187 | Chr02 | - | 669 | 8.08 | 71429.66 |
| MLbHLH168 | ML28922 | Chr14 | - | 97 | 8.43 | 10692.99 |
| MLbHLH169 | ML29183 | Chr01 | - | 96 | 9.46 | 10756.08 |
| MLbHLH170 | ML31130 | tig00000008 | - | 350 | 7.23 | 38440.45 |
| MLbHLH171 | ML32844 | Chr05 | + | 300 | 5.12 | 31533.73 |
